# Di- and tri-component spinel ferrite nanocubes: synthesis and their comparative characterization for theranostic applications†

**DOI:** 10.1039/d1nr01044a

**Published:** 2021-08-03

**Authors:** Niccolò Silvestri, Helena Gavilán, Pablo Guardia, Rosaria Brescia, Soraia Fernandes, Anna Cristina S. Samia, Francisco J. Teran, Teresa Pellegrino

**Affiliations:** Istituto Italiano di Tecnologia Via Morego 30 16163 Genova Italy Teresa.Pellegrino@iit.it; IREC-Catalonia Institute for Energy Research, Jardins de les Dones de Negre 1 Sant Adria de Besos 08930 Barcelona Spain; Department of Chemistry, Case Western Reserve University 10900 Euclid Avenue Cleveland OH 44106 USA; iMdea Nanociencia, Campus Universitario de Cantoblanco 28049 Madrid Spain; Nanobiotecnología (iMdea-Nanociencia), Unidad Asociada al Centro Nacional de Biotecnología (CSIC) 28049 Madrid Spain

## Abstract

Spinel ferrite nanocubes (NCs), consisting of pure iron oxide or mixed ferrites, are largely acknowledged for their outstanding performance in magnetic hyperthermia treatment (MHT) or magnetic resonance imaging (MRI) applications while their magnetic particle imaging (MPI) properties, particularly for this peculiar shape different from the conventional spherical nanoparticles (NPs), are relatively less investigated. In this work, we report on a non-hydrolytic synthesis approach to prepare mixed transition metal ferrite NCs. A series of NCs of mixed zinc-cobalt-ferrite were prepared and their magnetic theranostic properties were compared to those of cobalt ferrite or zinc ferrite NCs of similar sizes. For each of the nanomaterials, the synthesis parameters were adjusted to obtain NCs in the size range from 8 up to 15 nm. The chemical and structural nature of the different NCs was correlated to their magnetic properties. In particular, to evaluate magnetic losses, we compared the data obtained from calorimetric measurements to the data measured by dynamic magnetic hysteresis obtained under alternating magnetic field (AMF) excitation. Cobalt-ferrite and zinc-cobalt ferrite NCs showed high specific adsorption rate (SAR) values in aqueous solutions but their heating ability was drastically suppressed once in viscous media even for NCs as small as 12 nm. On the other hand, non-stoichiometric zinc-ferrite NCs showed significant but lower SAR values than the other ferrites, but these zinc-ferrite NCs preserved almost unaltered their heating trend in viscous environments. Also, the presence of zinc in the crystal lattice of zinc–cobalt ferrite NCs showed increased contrast enhancement for MRI with the highest *T*_2_ relaxation time and in the MPI signal with the best point spread function and signal-to-noise ratio in comparison to the analogue cobalt-ferrite NC. Among the different compositions investigated, non-stoichiometric zinc-ferrite NCs can be considered the most promising material as a multifunctional theranostic platform for MHT, MPI and MRI regardless of the media viscosity in which they will be applied, while ensuring the best biocompatibility with respect to the cobalt ferrite NCs.

## Abstract 2

Mixed transition metals ferrites nanocubes are here prepared. Their magnetic properties are evaluated to assess their applicability as theranostic tools for magnetic hyperthermia treatment, magnetic resonance imaging and magnetic particles imaging.

## Graphical abstract

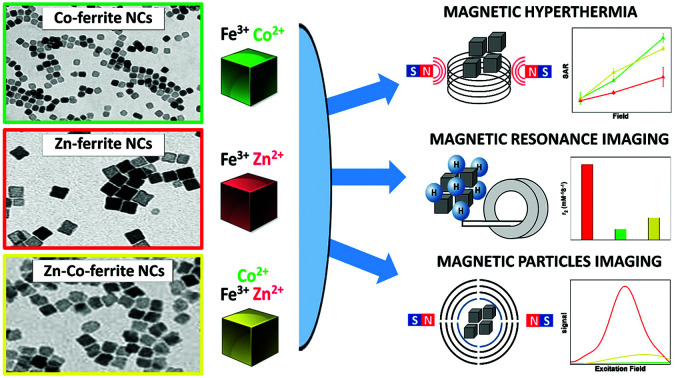

## Introduction

Iron oxide nanocubes (IONCs) are promising materials in the biomedical field.^1–4^ A clear example are magnetosomes,^5^ which are naturally produced liposome coated nanocrystals (30–120 nm) of magnetite with a cubic/cuboid shape that are often organized in chain structures. Their high chemical and crystal purity and narrow size distribution, positively affect their *T*_2_-relaxivity (as negative MRI contrast agents^6^), MPI performance^7^ and SAR value for MHT.^1,8^ Perhaps one of the main drawbacks is their bacteria derivation, which limits their use in human applications due to possible immune-response reactions. On the other hand, engineered IONCs that are chemically synthesized using thermal decomposition methods display similar qualities and excellent magnetic performance similar to that of magnetosomes.^9^ Moreover, the synthetic route offers the possibility of fine tuning the size in a large range (from 14 to 80 nm) and the composition of the NCs. Ferrites (such as IONPs) of the general formula MFe_2_O_4_, display a spinel structure (both normal and inverse) where M could be any of the divalent transition metal ions (Fe^2+^, Co^2+^, Zn^2+^, Mn^2+^, *etc*.). The spinel ferrite structure corresponds to a face-centered cubic arrangement of oxygen atoms, with M^2+^ and Fe^3+^ occupying tetrahedral (A) sites and Fe^3+^ occupying also octahedral (B) sites. For instance, stoichiometric zinc-ferrite (Zn-ferrite) has a spinel structure with Zn^2+^ ions occupying the A sites and Fe^3+^ ions occupying the B sites.^10,11^ The doping of iron oxide (magnetite) ferrites with other metal ions (as Co^2+^, Zn^2+^, Mn^2+^) offers an alternative way to improve the physical properties of magnetic ferrite NPs and thus their usefulness in bioapplications.^12^ For instance, starting from Fe_3_O_4_ (magnetite), Fe^2+^ cations can be partially substituted by divalent cations such as Co^2+^ or Zn^2+^ leading to Zn-ferrite and cobalt-ferrite (Co-ferrite) NPs.^10,11,13^ Co-ferrite has gained attention because of its strong magneto-crystalline anisotropy, moderate high saturation magnetization, and high coercivity.^14–16^ For example, Co-ferrite NCs synthesized by non-hydrolytic methods were proven to have higher SAR values than IONCs prepared in aqueous media: indeed, by finely tuning the NC size (from 15 to 27 nm) and the cobalt stoichiometry (from *x* = 0.1–0.7 in Co_*x*_Fe_3−*x*_O_4_) it was found that the cobalt content governs the heating properties of the final nanocubes.^16,17^ However, increasing the viscosity of the dispersion media reduces SAR values due to restriction of the Brownian magnetic relaxation.^18^ Moreover, in an *in vivo* preclinical study on Co-ferrite NCs at the primary tumor site, the authors have shown how it is possible to exploit the intrinsic toxicity of cobalt ions, slowly released upon NC degradation in combination with their very mild hyperthermia and their unique NC alignment feature under MHT as multifunctional platforms to completely suppress tumor growth.^19,20^ Alternatively, the Zn-ferrite composition may be used as a more biocompatible formulation than Co-ferrite. The Food and Drug Administration (FDA) sets the reference daily intake (RDI) doses for Fe and Zn at 18 and 15 mg day^−1^ much higher than that of Co ions (5–8 μg day^−1^).^21^ Spherical non-stoichiometric Zn-ferrite NPs meaning Zn_*x*_Fe_3−*x*_O_4_ with *x* < 1, hereafter referred to with the general name of Zn-ferrite NPs/NCs, have been extensively studied as contrast agents in MRI.^22–26^ Despite their poor size and shape distributions, good relaxivity values and improved detection sensitivity were achieved.^25,27^ Noh *et al.* reported an interesting work on 18 nm Zn_0.4_Fe_2.6_O_4_ NCs exhibiting high *M*_s_ (165 Am^2^ kg^−1^) and a maximum SAR of 1860 W g^−1^ under AMF excitation at a field frequency of 500 kHz and amplitude of 37.4 kA m^−1^.^17^ Moreover, these NCs showed good performance as MPI tracers.^7^ The clear improvement of these nanocube-shaped NPs over the previously reported spherical NPs is partially ascribed to a higher crystallinity and partially to the less spin canting effects occurring at the surface of the NCs with respect to the spherical NPs.^28^ Beyond this work, some studies reported double ion substitution in spherical shaped IONPs.^29–32^ For instance the mixed nanoparticle formulation Co_1−*x*_Zn_*x*_Fe_2_O_4_ could improve the chemical stability, corrosion resistivity, magneto–crystalline anisotropy, and magnetostriction and magneto–optical properties.^33–35^ Ben Ali *et al.* observed a significant decrease in coercivity from 2000 Oe to 170 Oe with increasing zinc substitution in Co_1−*x*_Zn_*x*_Fe_2_O_4_ NPs,^36^ which was attributed to the reduction of the magneto-crystalline anisotropy.^37^

They also reported a *M*_s_ of about 61 Am^2^ kg^−1^ for CoFe_2_O_4_ NPs of 11.7 nm (*versus* 81 Am^2^ kg^−1^ for bulk CoFe_2_O_4_),^38^ while the introduction of zinc ions brought an increase in saturation magnetization up to 75 Am^2^ kg^−1^ for Co_0.7_Zn_0.3_Fe_2_O_4_.^36^ This result is also supported by the research findings of other groups.^39,40^ The goal of this study is to synthesize high quality cubic shaped NPs with tunable Co and Zn stoichiometry using a non-hydrolytic synthetic approach. Starting with high quality Co-ferrite NCs and by partially substituting some of the cobalt by zinc ions, high quality zinc-cobalt mixed ferrite NCs were obtained. By exploiting a combination of experimental parameters such as Schlenk line pressure during the degassing step, volume of the reaction flask, and nitrogen flow rate, size control of Co-ferrite NCs smaller than 15 nm and up to 8 nm was reported; a size range that minimizes inter-particle interactions while maintaining high crystallinity. By changing the metal precursor mixture under similar reaction conditions, zinc-cobalt mixed ferrite NC samples were also obtained and compared to the Co-ferrite and Zn-ferrite NCs for their performance in MHT, MRI, and MPI to evaluate composition-dependent features. Finally, the cytotoxicity of Co and Zn-ferrite NCs was also evaluated using *in vitro* cell viability assays.

## Results and discussion

### Synthesis of cobalt ferrite NCs: control over size and shape

**Fig. 1:**
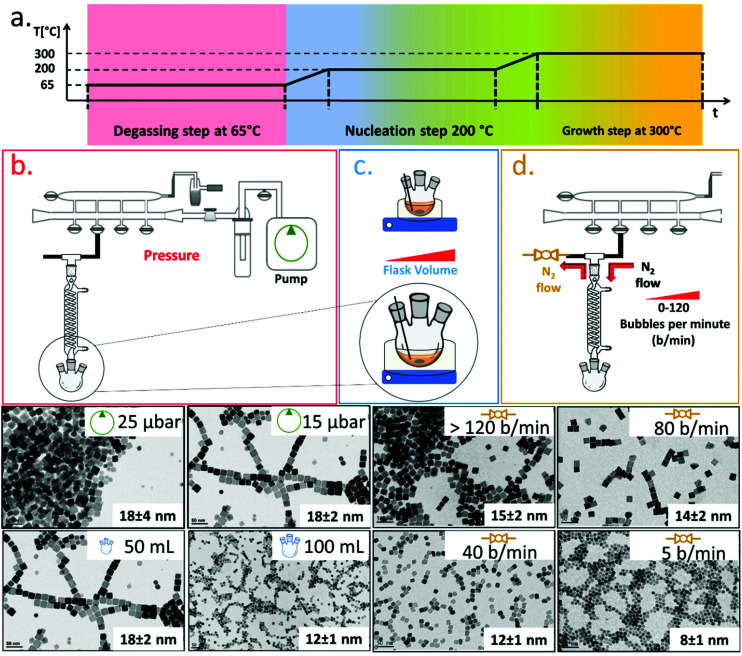
Synthesis sketch and main parameters involved in the size and shape control of Co-ferrite NCs. (a) Scheme of the heating ramps with the three main steps highlighted: the degassing (red), the nucleation (blue) and the growth (orange) of the NCs. Effect of (b) pressure achieved in the degassing step of the reaction (25–15 μbar); (c) volume of the flask (50–100 mL) (d) nitrogen flow (5–40–80–120 bubbles per minute). The pressure improves the size distribution, passing from a standard deviation of ±4 nm at 25 μbar to ±2 nm at 15 μbar. The flask volume and the nitrogen flow affect the nanocrystal size more enabling to obtain NCs with an edge size from 8 to 18 nm, as evidenced in the BF-TEM images below the sketches.

To synthesize ferrite NCs, the starting protocol used here refers to a previous published procedure by our group^41^ consisting of three-steps: (1) a degassing step at 65 °C under reduced pressure that ensures dissolution of the reagents and guaranteed oxygen and water-free atmosphere conditions; (2) a nanocrystal nucleation step at 200 °C under nitrogen gas; and (3) a growth step at 300 °C under a nitrogen gas atmosphere. We started with the protocol to obtain Co-ferrite NCs of 18 nm in cube edge.^16^ For this synthesis, 0.5 mmol Co(ii) acetylacetonate (Co (acac)_2_), 1 mmol Fe(iii) acetylacetonate (Fe (acac)_3_), 6 mmol decanoic acid (DA), 12 ml of squalane (SQ) and 13 mL of dibenzylether (DBE) were mixed and the three-step heat profile was applied (Fig. 1a). First, the effect of the Schlenk line pressure during the degassing step was evaluated. For a standard degassing step at 65 °C a usual 25–50 μbar is achieved leading to NCs of 18 ± 4 nm average edge size, as measured by bright field transmission electron microscopy (BF-TEM). On the other hand, by achieving a better vacuum with a more powerful vacuum pump, we reached a 15 μbar pressure and a narrower NC size distribution was obtained corresponding to 18 ± 2 nm (Fig. 1a). Likely, the presence of oxygen or moisture during the reaction may interfere with the growth of the NCs and therefore a more efficient degassing step is required for the control of the size distribution. Next, having fixed the final degassing pressure at 15 μbar, the second parameter that was changed was the reaction flask volume. Indeed, heat distribution in the reaction mixture plays a key role on the NC nucleation and the growth step, and to burst the nucleation process, a homogeneous and fast heat transfer from the heating mantle to the reaction solution should be guaranteed. Two syntheses were run in parallel in which the reaction mixture volume was kept constant (25 mL) at the same composition as described above but this same amount was placed either in a flask of 50 mL, as usually done, or choosing a flask with a bigger volume capacity of 100 mL. When comparing the product of the two syntheses, Co-ferrite NCs of 12 ± 1 nm were obtained upon using the flask of 100 mL capacity, instead of the 18 ± 2 nm NCs usually obtained when using the 50 mL flask (Fig. 1b). Likely, a flask with larger volume capacity maximizes the surface contact between the solution and the outer heating mantle source; hence, the heat distribution and the temperature remain more controlled during the reaction synthesis process. This experimental set-up seems to facilitate the nucleation as smaller NCs were obtained.

As a third parameter, we have considered the flow rate of the nitrogen gas that is used to purge the reaction flask during the NC synthesis. Usually, the nitrogen pressure at the inlet of the Schlenk line is set at 2 bar and during the nucleation and growth steps does not change but, so far, the nitrogen flow rate has not been properly controlled. The nitrogen flow into the reaction flask can be changed by controlling the nitrogen bubbles per minute (b per min) observed in the oil bubbler, positioned at the outlet of the flask (Fig. 1). In this set of reactions, having set the degassing pressure to 15 μbar and the volume flask at 100 mL, by drastically decreasing the nitrogen flow rate from 120 b per min to 5–10 b per min, it was possible to reduce the size of the Co-ferrite NCs from 15 nm to 8 nm (Fig. 1c). Also, by choosing intermediate nitrogen flow rates, at 80 or 40 b per min, NCs with a mean size of 14 ± 2 nm and 12 ± 1 nm were obtained, respectively. It is known that in this synthesis, one of the two solvents, dibenzylether (DBE), is not inert but it decomposes at 295 °C and especially in the presence of carboxylic acid, it generates volatile by-products (benzaldehyde, benzyl-alcohol and toluene), with benzaldehyde having an important contribution to the cubic shape control.^1,42^ Here, it is likely that at a low nitrogen flow rate (5–10 b per min), the purging of these by-product species is slower than that occurring upon exposure to nitrogen flow at 120 b per min, and their longer persistence in the reaction mixture favours the nucleation of the nanocrystals and helps the cubic shape formation during the growth step.

### Synthesis of mixed ferrite NCs: control over the composition

**Fig. 2:**
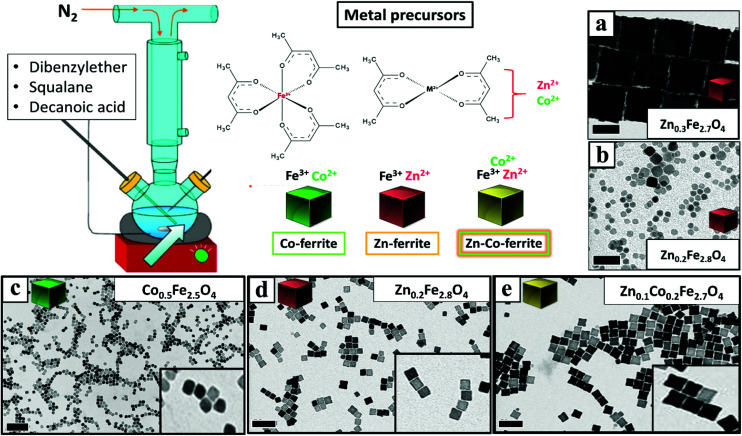
BF-TEM images of mixed ferrite NCs: (a) Zn_0.3_Fe_2.7_O_4_ NCs 65 ± 5 nm; (b) Zn_0.2_Fe_2.8_O_4_ NCs 9 ± 3 nm; (c) Co_0.5_Fe_2.5_O_4_ NCs 12 ± 1 nm; (d) Zn_0.2_Fe_2.8_O_4_ NCs 12 ± 1 nm; (e) Zn_0.1_Co_0.2_Fe_2.7_O_4_ NCs 15 ± 1 nm. These NCs were prepared by using different feed ratios of Zn/Co metal precursors [Zn(acac)_2_ and Co(acac)_2_], (a–b–d) only Zn(acac)_2_, (c) only Co(acac)_2_, and (e) Zn(acac)_2_ and Co(acac)_2_. In all synthesis the total amount of metal precursors was fixed at 1.5 mM with Fe(acac)_3_ fixed at 1 mM. The DA surfactant was fixed at (a) 8 mmol or (b) 6 mmol or (c) 7 mmol. All the other reaction parameters were identical (100 mL flask, nitrogen flow at 40 b per min). Scale bars (in black) indicate 50 nm in all images.

Once having set the above crucial parameters for the synthesis of NCs smaller than 15 nm (degassing final pressure 15 μbar, 100 mL flask and nitrogen flow at 40 b per min), we aimed at changing the composition of the NCs from that of Co-ferrite to others such as that of Zn-ferrites. First, the complete substitution of the Co precursor with the Zn precursor led to NCs with a marked increase in size, passing from 12 nm for the Co-ferrite to 65 nm for that of Zn-ferrite (Fig. 2a and Fig. S1†). This is likely attributed to the slower decomposition rate of the Zn precursor in comparison to the cobalt precursor, in the reaction mixture.^43^ Indeed, when looking at the decomposition temperature measured by thermogravimetric analysis of Zn(acac)_2_, it was found that it is higher (300 °C) than that of Co(acac)_2_ (250 °C).^44,45^

To reduce the Zn-ferrite NC size, the amount of surfactant (DA) in the synthesis was increased from 6 to 7 mmol (corresponding to a surfactant to metal Fe + Zn molar ratio of 4.7) while keeping the rest of the precursors as in the first synthesis attempt for Zn-ferrite (1 mmol Fe (acac)_2_, 0.5 mmol Zn(acac)_2_, 13 mL of DBE and 12 mL of SQ). As expected, the increase in surfactant promotes nucleation and the presence of more nuclei consumes more precursors leading to smaller and well-defined NCs of 12 ± 1 nm in cube edge and with a stoichiometry of Zn_0.2_Fe_2.8_O_4_ (Fig. 2d).^46^ Moreover, in the attempt to increase the Zn content in the NCs, the Zn precursor was doubled (1 rather than 0.5 mmol as in the synthesis of Fig. 2d while maintaining the same reaction conditions), but the final Zn stoichiometry remained at 0.2 even if the NCs obtained were very similar in terms of cube-edge size (*ca.* 12 nm, data not shown). It is worth noting that when the same synthesis was repeated by adding an even higher DA surfactant amount (8 mmol), smaller NPs (9 ± 3 nm) were obtained but being too small, their cubic shape was not well-defined (Fig. 2b). These first set of results suggest that the decomposition kinetics of the Zn and Co precursors are rather different, hence, the synthesis parameters for the synthesis of Zn–Co-ferrite NCs needed to be re-adjusted.

To this aim, we set the same synthesis parameters used for the synthesis of Co-ferrite NCs, but both Zn(ii) and Co(ii) acetylacetonate precursors were added. For the mixed ferrites the overall amount of cobalt metal ions (0.5 mmol) was replaced with the sum of Zn + Co metal precursors (0.25 mmol Zn and 0.25 mmol Co) while the iron precursor amount (1 mmol) was kept constant. Also in this case the DA surfactant (6 mmol) to metal precursor (iron + cobalt + zinc) molar ratio was kept to 4 : 1. Under these conditions NCs of 15 ± 1 nm with a well-defined shape and with a mixed composition (Zn_0.1_Co_0.2_Fe_2.7_O_4_) were obtained (Fig. 2e and Fig. S1†). It should also be noted that the Zn stoichiometry obtained is well below 1 typical of pure Zn-ferrite (ZnFe_2_O_4_) and is much lower than that of Co.

**Fig. 3:**
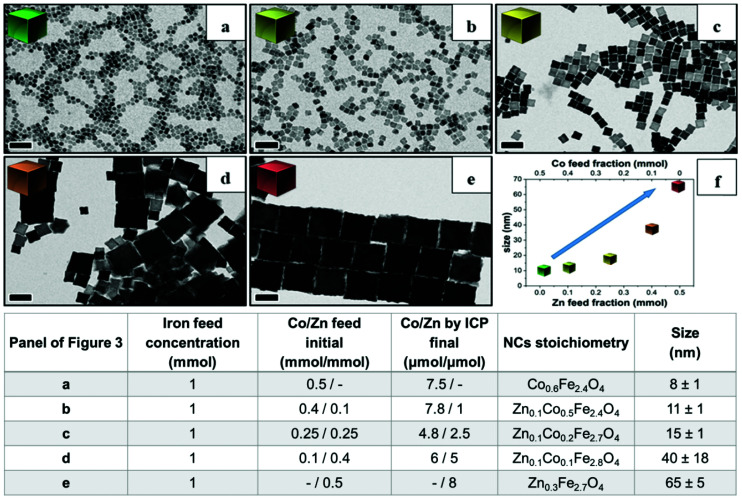
BF-TEM images of mixed Zn–Co-ferrite NCs obtained by varying the Co/Zn feed molar ratio in the reaction mixture. Table summarizing the Fe, Co/Zn feed ratio and the relative stoichiometry and size of the obtained NCs as measured by elemental analysis: (a) only Co precursor (no Zn), Co/Zn at (b) 0.4 mmol/0.1 mmol, (c) 0.25 mmol/0.25 mmol and (d) 0.1 mmol/0.4 mmol. (e) Only Zn-precursor (no Co) (f) a graph summarizing the NC size trend as a function of Co and Zn precursors. Scale bars indicate 50 nm in all images.

Next, to change the relative Zn to Co stoichiometry, a set of experiments were performed by changing the starting amounts of Zn(acac)_2_ and Co(acac)_2_ in the feeding reaction pot, while consistently keeping the amount of Fe(acac)_3_ (1 mmol), the DA amount (6 mmol) and the rest of the reaction parameters constant. Zn-Co-ferrite NCs at different stoichiometries could be obtained, however, the size of the NCs was markedly affected, and NCs in the range from 8 to 65 nm were obtained, depending on the precursor amounts (Fig. 3 and Fig. S2†). At an increased amount of Zn precursors in the feeding pot, the size of the NCs tended to increase, as the Zn precursors hindered the nucleation while promoting crystal growth. When using an equimolar amount of Zn and Co (a Co : Zn feeding ratio 1 : 1, 0.25 : 0.25 mmol), the amount of Co was double that of Zn in the final NCs as measured from ICP-OES elemental analysis. The same trend was found for other mixed compositions : when starting with Co : Zn feeding ratio of 0.4 : 0.1 or 0.1 : 0.4 mmol, the Co : Zn ratio in the NCs was 7.8 : 1 or 6 : 5 μmol, respectively.^2^ Therefore, in all the investigated mixed Co–Zn-ferrite NCs, the amount of Zn in the final NCs was always lower than that of Co. These data again confirm that the decomposition of the Co precursor is certainly faster than that of the Zn precursor, thus favouring the incorporation of more Co than Zn into the growing mixed ferrite NCs.^44,45^

**Fig. 4:**
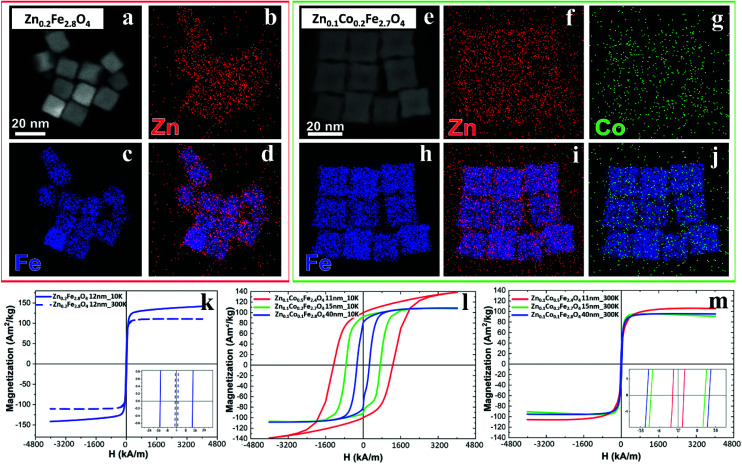
Compositional analysis by EDS and magnetization *vs.* applied magnetic field curves for different ferrite samples. (a and e) High-angle annular dark-field STEM (HAADF-STEM) images of NCs for (a) Zn_0.2_Fe_2.8_O_4_ and (e) Zn_0.1_Co_0.2_Fe_2.7_O_4_ samples and (b–d, f–j) corresponding STEM-EDS elemental maps for single element Fe (blue), Co (green) and Zn (red) and merged in (d, i and j) for Zn_0.2_Fe_2.8_O_4_ (red squared box) and Zn_0.1_ Co_0.2_Fe_2.7_O_4_ (green squared box) respectively. M–H magnetization curves at 10 and 300 K for (k) Zn-ferrite NCs 12 ± 1 nm; and for Zn–Co-ferrite NCs at different sizes at 10 K (l) and 300 K (m).

The cation concentration for the Zn-ferrite (Zn_0.2_Fe_2.8_O_4_) and Zn-Co-ferrite (Zn_0.1_Co_0.2_Fe_2.7_O_4_) NCs were analyzed by energy-dispersive X-ray spectroscopy (EDS) combined with scanning TEM mode (STEM-EDS) (Fig. 4a–j and S3†). For both samples, the average cation concentration in the NCs measured by EDS matched very well the composition measured *via* elemental analysis using ICP (Table S1†).

Next, X-ray diffraction (XRD) analysis indicates that the positions and relative intensities of all diffraction peaks matched very well to the standard diffraction patterns for inverse spinel Fe_3_O_4_ powder (Fig. S4†). Moreover, high resolution TEM (HRTEM) analyses of individual NCs confirmed the expected {100} faceting of the NCs and match the phase evaluated from XRD analysis, with no significant changes in lattice parameters along the NCs’ volume (Fig. S5†).

The same samples were characterized to investigate their magnetic properties, which was obtained by recording the hysteresis loops with finite coercive fields (H_c_) and the temperature dependent magnetization curves (Fig. 4k–m). The 12 ± 1 nm Zn-ferrite NC (Zn_0.2_Fe_2.8_O_4_) sample showed a significantly low coercive field and high saturation magnetization at both 300 K and 10 K, being superparamagnetic at 300 K, in accordance with the literature.^7,47^ The inclusion of cobalt ions into the Zn-Co-ferrite NC of the 11 nm sample (Zn_0.1_Co_0.5_Fe_2.4_O_4_) also showed superparamagnetic behaviour at 300 K, but the H_c_ drastically increased at 10 K (1600 kA m^−1^), so other magnetic structures may have affected the overall magnetic features. In general, such a behaviour is ascribed to the formation of core–shell structures,^16,48^ which may lead to the hypothesis that the NCs present an iron-rich core and a Zn/Co-rich shell ferrite structure. This hypothesis however is not fully supported by STEM-EDS elemental mapping of the cation distribution over the NCs (Fig. 4a–j), which does not show a clear enrichment in Zn and Co at the surface of the NCs. The Zn/Co surface enrichment may however be of a few percent compared to the overall content and therefore too low to be assessed by this technique (see Table S1†). Moreover, in the STEM-EDS maps the Co signal does not appear clearly localized within the NCs (Fig. 4g), while the Zn signal is more intense within the NCs. This is also due to the fact that the highest intensity peak of Co (K_α_, centered at 6.93 keV) could not be used for the maps because of its partial overlap with the Fe K_β_ peak (centered at 7.06 keV, see Fig. S3†).

### Characterization of ferrite NCs for magnetic hyperthermia, MRI, and MPI applications

To evaluate their MHT, MRI, and MPI performance, the as-synthesized ferrite NCs were transferred from chloroform to water by using an amphiphilic polymer, poly(maleic anhydride-*alt*-1-octadecene) (PMAO), and following a well-established polymer coating procedure (see Experimental section).^49^ The Zn-ferrite NCs and mixed Zn-Co-ferrite samples’ properties were compared to those of Co-ferrite NCs (sample in Fig. 2) by choosing a set of samples that had a very similar TEM NCs size (*ca.* 15 nm). The polymer coating protocol provided NCs well-dispersed in water with a monomodal hydrodynamic peak as measured by dynamic light scattering (DLS), with a polydispersity index (PDI) below 0.15 for all the samples as an indication of their good dispersion in aqueous media (Table S2†). Indeed, the hydrodynamic intensity peaks for Co-ferrite, Zn-ferrite and Zn-Co-ferrite NCs were centered at 32 ± 12 nm (PDI 0.14), 57 ± 20 nm (PDI 0.12) and 36 ± 14 nm (PDI 0.13), respectively (Table S2†), indicating that the NCs have larger hydrodynamic sizes (*D*_h_) than the corresponding TEM core size, which is in accordance with being coated with the hydrated polymer shell in water, but still they have quite a small size and PDI below 0.2 indicating the presence of individually coated NCs in solution.

#### Ferrite NCs for MHT

**Fig. 5:**
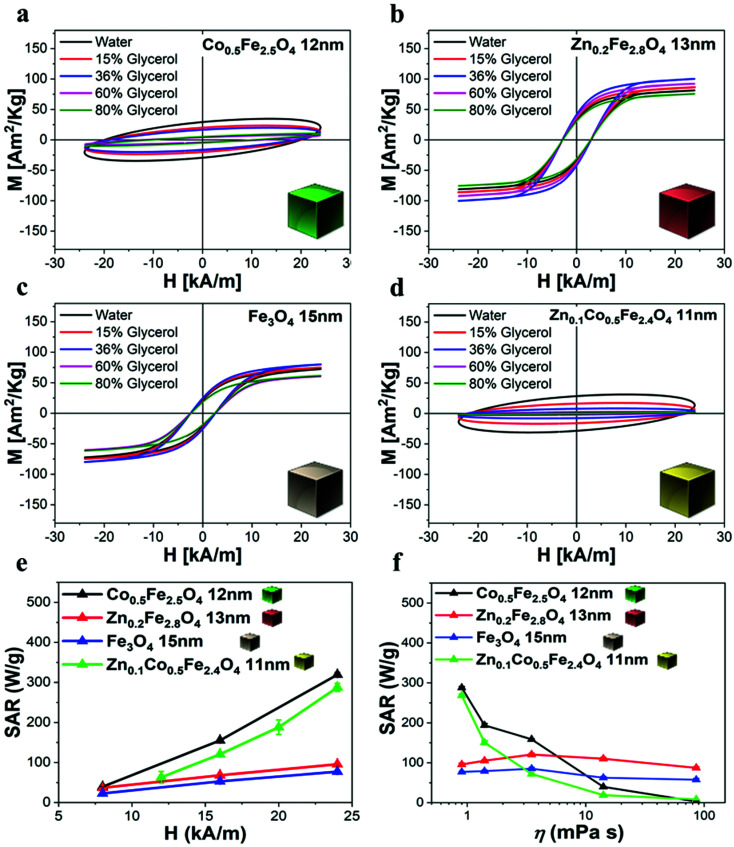
AC hysteresis loop characterization and SAR graphs of different ferrite samples in water and in viscous media. Magnetic AC hysteresis loop viscosity dependence measurements of (a) Co-ferrite NCs, (b) Zn-ferrite NCs, (c) IONCs and (d) Zn-Co-ferrite NCs under AMF (100 kHz and 24 kA m^−1^). The percentages of glycerol are 0–15–36–60–80% (corresponding to 0.9, 1.4, 3.5, 14 and 85.9 mPa s) in a water solution at 2 g L^−1^ of magnetic elements. (e) SAR values calculated by AC magnetometry for the different water dispersed ferrite NC samples (2 g L^−1^ of magnetic elements) of comparable sizes at 100 kHz and 8, 16 and 24 kA m^−1^. (f) SAR values calculated by AC magnetometry measurements at increasing glycerol percentage and thus at different media viscosity under AMF (24 kA m^−1^) at 100 kHz. Lines are a guide to the eye. The error bars are not visible, with the standard deviation less than 2% of the measured value in most of the shown data.

To evaluate the performance of the ferrite NCs as heating mediators for MHT, the SAR values of the polymer coated NCs in water were evaluated by inductive measurements using AC magnetometry to record the dynamic hysteresis loops in the frequency range from 100 kHz up to 300 kHz, while varying the field intensity from 4 up to 24 kA m^−1^ (Fig. 5). For these measurements, for the different ferrites (Co-ferrite NCs, Zn-ferrite NCs, and Zn-Co-ferrite NCs) the concentration of the samples in terms of the total metal amount (Co + Fe or Zn + Fe or Zn + Co + Fe, respectively) was fixed at 2 g L^−1^. The dynamic hysteresis loop (DHL) curves (Fig. 5) and the corresponding SAR values, calculated as SAR = *A*·*f*,^50^ where *A* is the area of the magnetic loop and *f* the field frequency (*i.e.* 100 kHz) and under different field intensity values (from 12 to 32 kA m^−1^), revealed that, Co-ferrite and Zn-Co-ferrite NCs showed the highest opening of the DHL which, in turn, corresponds to the highest SAR values (up to a maximum of about 300 W g^−1^ at the of 24 kA m^−1^) (Fig. 5). At a high frequency (300 kHz), the highest SAR values recorded were those measured by the Zn-ferrite NCs (maximum of about 500 W g^−1^ at 24 kA m^−1^) (Fig. S6†). To mimic the biological environment, the dynamic hysteresis loops were also measured on NCs dispersed in viscous media using increasing glycerol/water mixtures with a gradual increase of the glycerol percentage. In this case, the AMF was set at 100 kHz and 24 kA m^−1^ to keep the treatment conditions below the safe clinical limits (*H*·*f* < 5 × 10^9^ A m^−1^ s^−1^).^51^ The DHL areas of Zn-ferrite NCs and IONCs remained unchanged even at increased values of viscosity (Fig. 5d and e), while those of cobalt-based ferrite NCs decreased with increasing media viscosity, thus reducing the heating ability (Fig. 5d and g). As such, Zn-ferrite NCs and IONCs of 13 and 15 nm in cube edge lengths, respectively, were not affected by viscosity changes. The Zn-ferrite NC samples showed nearly SAR-viscosity independent behaviours revealing that the Néel magnetic relaxation process was the dominant mechanism over Brownian relaxation for Zn-ferrite NCs with sizes below 15 nm. In comparison, the SAR values drastically dropped almost to 0 at high viscosities for all the ferrites that contained Co, even at a low fraction of Co as for the case for the Co_0.5_Fe_2.5_O_4_ (12 nm) and Zn_0.1_Co_0.5_Fe_2.4_O_4_ NCs (11 nm) samples. This behaviour was already observed in a previous work but for larger Co-ferrite NCs (21 nm).^18^ Moreover, likely due to the low content fraction of Zn in the mixed Zn_0.1_Co_0.5_Fe_2.4_O_4_ NCs, no significant differences in the hysteresis loop areas were observed with respect to the Co_0.5_Fe_2.5_O_4_ NCs. Possibly, the prevalence of Brownian relaxation processes on the heating mechanism rather than the Néel contribution for the Co-based NCs determines the SAR behaviour observed when increasing the media viscosity.^18^

It is worth noting that for all the aqueous samples, the SAR data obtained by AC magnetometry measurements are fully in accordance with the ones obtained from calorimetric measurements when using similar sample volumes and field conditions (Table 1 and Fig. S7†). The field conditions of the calorimetry and AC magnetometry equipment were close enough in values but not identical according to the instrument available (100 kHz and 16 kA m^−1^ for the AC magnetometer and 100 kHz and 20 kA m^−1^ for the calorimetric device).

**Table 1:** Comparison of SAR values of different aqueous ferrite samples measured by AC magnetometry and calorimetric measurements

Sample	SAR magnetic measurements (W g^−1^) *f*: 100 kHz, *H*: 16 kA m^−1^	SAR calorimetric measurements (W g^−1^) *f*: 100 kHz, *H*: 20 kA m^−1^
Fe_3_O_4_ NCs, 15 nm	53 ± 1	69 ± 14
Co_0.5_Fe_2.5_O_4_ NCs, 12 nm	155 ± 2	151 ± 9
Zn_0.2_Fe_2.8_O_4_ NCs, 13 nm	68 ± 2	93 ± 7
Zn_0.1_Co_0.5_Fe_2.4_O_4_ NCs, 11 nm	121 ± 2	188 ± 18

#### Ferrite NCs and their performance in MPI and MRI

**Fig. 6:**
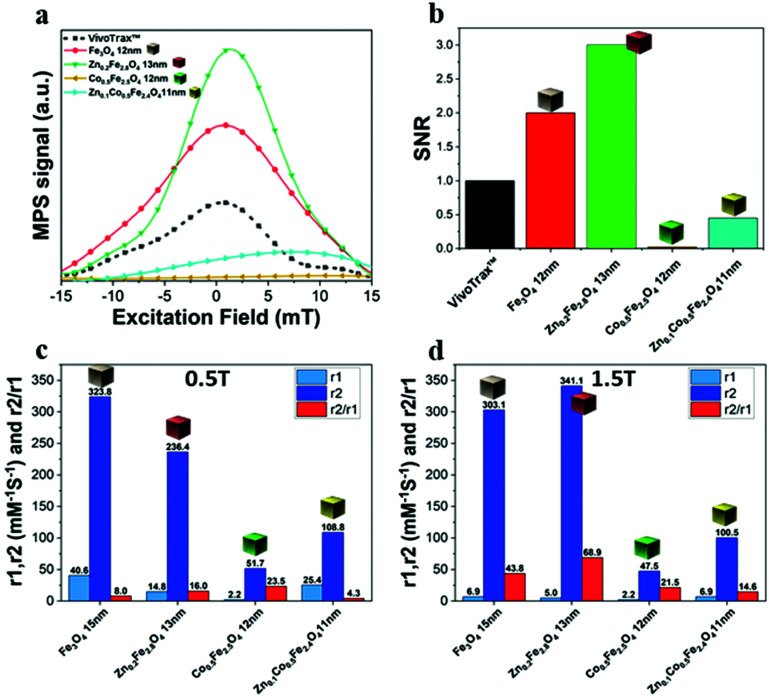
The normalized point spread function (PSF) (a) and the signal to noise ratio (SNR) (b) of ferrite NCs using as reference a commercial superparamagnetic iron oxide MPI tracer (VivoTrax™). Relaxation measurements of *r*_1_ = 1/*T*_1_, *r*_2_ = 1/*T*_2_ and *r*_2_/*r*_1_ ratio values recorded at 0.5 T (c) and 1.5 T (d) static field for the different ferrite samples.

The ferrite NCs were further evaluated for their MPI and MRI performance.^52^ MPI is indeed an emerging imaging technique for which ferrite NCs have thus far been only partially evaluated.^53–55^ Using a magnetic particle relaxometer, the NCs were exposed to a sinusoidal magnetic field (frequency 16.8 kHz, amplitude 16 kA m^−1^), which mimics the field free region (FFR) movement across the sample during an MPI experiment. The time dependent magnetization is inductively detected by a coil and gridded to the known instantaneous magnetic field value to generate the point spread function (PSF).^7^ Among the different ferrites in water, the most symmetric curve with the highest signal-to-noise ratio (SNR) and the narrower full-width at half-maximum (FWHM), type of signal,^52^ was achieved for the Zn-ferrite NCs, followed by the IONCs (Fig. 6a and b). Both samples had a higher SNR signal compared to a commercial MPI tracer (VivoTrax™): Zn-ferrite NCs and IONCs showed 3- and 2-fold higher SNR values compared to VivoTrax™, respectively (Fig. 6b). The Zn-Co ferrites had an SNR signal much lower than that of VivoTrax™ with a broad peak. On the other hand, the Co-ferrite NCs did not show any detectable signal. As demonstrated by Arami *et al.*,^56^ for spherical iron oxide NPs, it is likely that for NPs smaller than 15 nm the Néel relaxation process is the dominant mechanism determining the MPI response and that is also the same size range of our Zn-ferrite and IONCs (13 nm and 12 nm, respectively). For the Co-based ferrite NCs, Brownian relaxation is the dominant mechanism resulting in low SNR values. It is worth mentioning that the differences in MPI signal observed for these samples are in close agreement with the SAR values measured at different viscosities for the same samples, confirming that the most suitable samples for MPI and MHT are the ones showing a predominant Néel relaxation magnetic reversal process. The polymer shell and the media of the colloids may both play an important role in the signal generation affecting the Brownian relaxation of the NCs.^57,58^ Indeed, the magnetic particle signal recorded for the different as-synthesized ferrite NCs in chloroform and therefore stabilized by DA surfactants is higher in all the cases (Fig. S8†). In particular, the Co-based ferrite NCs showed a good signal in chloroform which was broader but comparable in intensity with that of VivoTrax™ (Fig. S8b–d†); the signals were drastically reduced once the DA stabilized-NCs were transferred into water and capped in the polymeric shell. The same ferrites were also evaluated as MRI contrast agents, by performing *T*_1_ and *T*_2_ relaxation time measurements. To achieve a high efficiency as a MRI contrast agent, it is crucial to perturb the longitudinal (*T*_1_) or transverse (*T*_2_) relaxation times (*r*_1_ = 1/*T*_1_ and *r*_2_ = 1/*T*_2_, respectively, and the corresponding *r*_2_/*r*_1_ ratio) of water proton spins in tissues at the lowest dose of contrast agents.^59^ Ferrite NPs, in particular IONPs, are well known as *T*_2_-contrast agents as they mostly affect the *T*_2_-signal of protons improving the dark signals of *T*_2_-weighted images in MRI.^6,41,60^ For the ferrite NCs in water, the relaxation time was recorded at 1.5 T and 0.5 T (Fig. 6c, d and Fig. S9†). The Co ferrite and the Zn-Co-ferrite NC samples showed the lowest *r*_2_ values.^61^ Interestingly, for the Zn-Co-ferrite NCs (Zn_0.1_Co_0.5_Fe_2.4_O_4_, 11 nm) the presence of Zn in addition to Co into the crystal, doubled its *r*_2_ relaxivity values (from 51.7 mM^−1^ s^−1^ to 108.8 mM^−1^ s^−1^ at 0.5 T and from 47.5 mM^−1^ s^−1^ to 100.5 mM^−1^ s^−1^ at 1.5 T) with respect to the Co-ferrite NC sample of a very similar size (11 nm *versus* 12 nm, respectively). The Zn-ferrite NCs show the highest *r*_2_ value at the 1.5 T field among all the measured samples. The best *T*_2_ relaxivity for this sample may be likely ascribed to the highest *M*_s_ at 300 K that is 120 Am^2^ kg^−1^ for Zn-ferrite NCs and 80 Am^2^ kg^−1^ for Zn–Co-ferrite NCs (Fig. 3) compared to 64 Am^2^ kg^−1^ for IONCs^1^ and 45 Am^2^ kg^−1^ for Co-ferrite NCs of a comparable size and cobalt content.^16^ Indeed, it is reported in the literature that the non-stoichiometric inclusion of non-magnetic metal ions (Zn) in an inverse spinel ferrite (Zn_*x*_Fe_3−*x*_O_4_), increasing the magnetic disorder in the crystal lattice, led to the magnetization saturation enhancement when *x* < 0.5 as it is for our samples (Zn_0.2_Fe_2.8_O_4_ and Zn_0.1_Co_0.5_Fe_2.4_O_4_).^25^ Finally, although all the samples are *T*_2_-contrast agents (the *r*_2_/*r*_1_ ratio for all the sample is higher than 14 at 1.5 T), the Zn-ferrite NCs revealed to have the highest value of the ratio *r*_2_/*r*_1_ (68.9) in comparison to Fe_3_O_4_ (43.8) and Co-based ferrites (21.5 and 14.6).

#### *In vitro* cytotoxicity of ferrite NCs

**Fig. 7:**
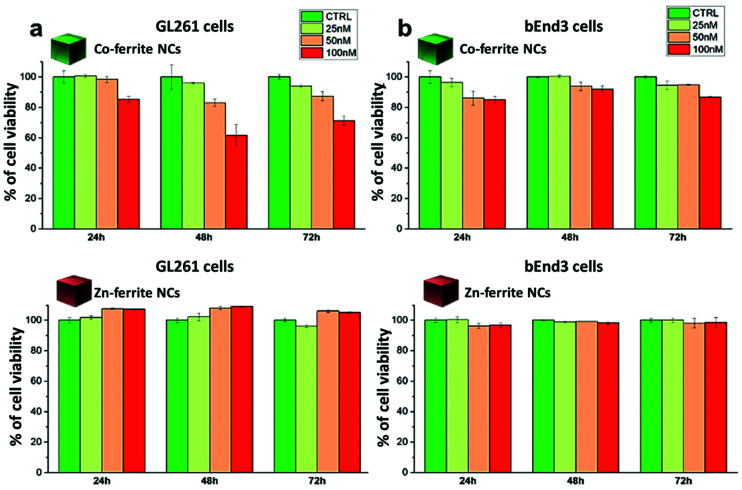
Cell viability analysis assessed by dead/live assay after exposure to Zn_0.2_Fe_2.8_O_4_ NCs (13 ± 1 nm) or Co_0.5_Fe_2.5_O_4_ NCs (12 ± 2 nm) of (a) murine glioblastoma cells (GL261) or of (b) murine vascular brain endothelial cells (bEnd3). The cells were incubated for 24, 48 and 72 hours at 37 °C using three different NC concentrations of 25, 50 and 100 nM (corresponding to 0.1, 0.2, 0.4 mg_Fe_ mL^−1^ and 0.02, 0.04, 0.08 mg_Co_ mL^−1^ for the Co-ferrite NCs; 0.14, 0.28, 0.56 mg_Fe_ mL^−1^ and 0.01, 0.02, 0.04 mg_Zn_ mL^−1^ for the Zn-ferrite NCs).

To exploit the ferrite NCs for biomedical applications it is important to assess their cytotoxicity. Viability assays were performed only for the Co_0.5_Fe_2.5_O_4_ (12 nm) and Zn_0.2_Fe_2.8_O_4_ (13 nm) samples, which were selected because of their promising magnetic performance for MHT, MPI and MRI compared to the other synthesized ferrite NC samples. Besides using the polymer coated NCs for these tests to ensure full NC stability in biological media, both types of NCs were additionally functionalized with diamine–polyethylene glycol (NH_2_–PEG–NH_2_, MW 2000) by standard EDC chemistry following a well-established procedure.^62^ The *in vitro* cytotoxicity assays were performed on a non-carcinogenic mouse brain endothelial (bEnd3) cell line and on a carcinogenic mouse glioblastoma (GL261) cells upon administration of NCs to the cell media.^63,64^ The tests were performed at three different NC concentrations (25, 50 and 100 nM) and at different incubation times (24, 48 and 72 h). After 24 h, there is a negligible percentage of cell death for both kinds of NCs and cell types (Fig. 7). However, the continuous exposure to Co-ferrite NCs, especially at the higher concentration investigated (100 nM), resulted in a reduction of cell viability over time (especially at 48 and 72 h). In contrast, the Zn-ferrite NCs did not show any significant signs of cell toxicity even at the highest administered NC concentration (100 nM). GL261, the tumor cell line, seemed more sensitive to the toxic effect of Co-ferrite NCs than the bEnd3 line, which can be likely attributed to the higher uptake of cancer cells with respect to non-tumorigenic cells (Fig. 7). Indeed, it is clear that there is dependence of the GL261 viability with the sample concentration, whereby a 60% viability was reached after 48 h when exposed to the highest concentration of NCs (100 nM), a toxic effect that persisted after 72 h exposure (Fig. 7a). These results were also supported by the visual image inspections acquired with an optical microscope on the cell culture dish (Fig. S10†). Comparing our viability results with previous studies in which mixed ferrite nanoparticles were employed is not straightforward, because often, the nanoparticles chosen for those studies differ in size, composition, shape and coatings, all parameters affecting nanoparticle behavior in cell media thus their cytotoxicity on chosen cancer cell lines.^65–68^ The cell viability findings with our Zn-ferrite NCs are in line with Zn-ferrite nanoparticles prepared by the electrochemical method, with a spherical shape, similar size (13 nm) and similar non-stoichiometry composition (Zn_0.5_Fe_2.5_O_4_), and administered at a similar nanoparticle dosage.^65^

For the Co-ferrite NCs, at 100 nM and after 72 h, the cell morphology looked altered, with membrane blabbing and clear signs of cell suffering. This is likely due not only to the intrinsic toxicity of the Co leaching from the Co-ferrite NCs but, also, to the NCs’ aggregation on their surface: some evident black spots were present on the cells and on the culture dish. On the other hand, the cells exposed to Zn-ferrite NCs under the same incubation conditions were in a healthy state showing a regular cell morphology although some Zn-ferrite NCs were also present as tiny aggregates on the cells.

## Conclusions

We have reported here the synthesis conditions to obtain ferrite nanocubes with a narrow size distribution and well-defined cubic shape below 15 nm and up to 8 nm cube edge. In particular, the effect of pressure during the degassing step, the temperature distribution during the nucleation and growth step, and the nitrogen flow bubbling rate were the parameters that were studied and have allowed to obtain such synthesis control on Co-ferrite NCs (Fig. 1). Moreover, the complete or partial substitution of the cobalt precursor with the zinc precursor has enabled to achieve Zn-ferrite or Zn-Co-ferrite NCs of high quality and in the same size range. In addition, the effect of Zn and Co feed metal precursor ratio on the composition of the resulting Zn-Co-ferrite NCs was also discussed (Fig. 2 and 3). The further wide range of magnetic characterization on the ferrite NCs has enabled us to compare their heating performance for MHT and their potential to be used as contrast agents for MRI and MPI applications (Fig. 4–6). For MHT, Co_0.5_Fe_2.5_O_4_ (12 nm) and Zn_0.1_Co_0.5_Fe_2.4_O_4_ (11 nm) NCs, in water, showed high SAR values (300 W g^−1^), but at low frequency (100 kHz and 24 kA m^−1^), when the media viscosity is increased, their heating ability is markedly suppressed (Fig. 5). Zn_0.2_Fe_2.8_O_4_ NCs with a size of 12 nm showed a high *M*_s_ value (about 120 Am^2^ kg^−1^ at 300 K) and superparamagnetic behaviours at 300 K; they displayed the best SAR performance among all the samples investigated especially in viscous media, achieving SAR values of 480 W g^−1^ at 300 kHz and 24 kA m^−1^ in water. Indeed, this Zn-ferrite sample showed a viscosity-independent SAR behaviour, and it does not lose its heating ability even in an 81% solution of glycerol and at a viscosity similar to those of the tumour environment, making these NCs very promising for *in vivo* MHT applications (Fig. 5). Furthermore, this sample showed the best performance among the different ferrite compositions investigated for acting as an MRI contrast agent with a *r*_2_ of 341 mM^−1^ s^−1^ at 1200 kA m^−1^ (1.5 T) and, also, the first candidate as a promising MPI tracer with the best point spread function and signal-to-noise ratio, that was 3-fold higher than the one recorded for VivoTrax™. It is noteworthy to mention that the Co-ferrite NC (12 nm) sample showed the lowest signal for both MRI (47.5 mM^−1^ s^−1^ at 1200 kA m^−1^) and MPI (almost no signal) measurements (Fig. 6). However, the partial substitution of the Co with Zn ions increased the NC performance, in particular Zn_0.1_Co_0.5_Fe_2.4_O_4_ (11 nm) NC samples had an SNR ratio 5-fold higher than that of Co_0.5_Fe_2.5_O_4_ (12 nm) as shown in Fig. 6b and *r*_2_ value 2-fold higher (100 mM^−1^ s^−1^ at 1200 kA m^−1^) than that of the Co-ferrite NCs as shown in Fig. 6d, making it useful for MPI and MRI applications. Finally, the biocompatibility of the synthesized Zn-ferrite NCs was shown *in vitro* on healthy brain endothelial murine cells (bEnd3) and brain tumour murine cells (GL261), confirming the suitability of such materials to be simultaneously employed as a heating mediator in MHT and as an imaging agent in MRI and MPI applications (Fig. 7). The proposed synthetic routes lead to metal doping of ferrites with tuneable magnetic properties suitable for biomedical applications.

## Materials

Iron(iii) acetylacetonate (Fe(acac)_3_, 99%) and decanoic acid (DA, 99%) were purchased from Acros. Cobalt(ii) acetylacetonate (Co (acac)_2_, 99%), zinc(ii) acetylacetonate hydrate (Zn (acac)_2_, 99%), poly maleic anhydride-*alt*-1-octadecene (PMAO), sucrose, agarose, dibenzylether (DBE, 98%), acetone (99.5%), chloroform (99.8%) and isopropanol (99.8%) were purchased from Sigma Aldrich. Squalane (SQ, 98%) was purchased from Alfa Aesar. Milli-Q water filtered through 0.22 μm pore size hydrophilic filters (18.2 MΩ cm) was supplied by a Milli-Q® Integral Water Purification System, and VivoTrax™ (Resovist®) licensed for distribution in the USA for pre-clinical MPI studies, was purchased from Magnetic Insight (Alameda, CA, USA). All chemicals were used as received. bEnd3 and GL 261 cell lines were purchased from ATCC, and Dulbecco's Modified Eagle's Medium (DMEM), fetal bovine serum (FBS), penicillin streptomycin (PS), pyruvate, non-essential amino acids (100×)(MEM) and glutamine were purchased from Sigma Aldrich.

### Synthesis of Fe_3_O_4_ nanocubes

Fe_3_O_4_ nanocubes (IONCs) were synthesized by thermal decomposition following an established procedure, with suitable modifications.^1^ The protocol relies on the synthesis of particles of 15 nm in size. In a 100 mL three-neck flask connected to a standard Schlenk line, 1.5 mmol (529 mg) Fe(acac)_3_ and 7 mmol (1205 mg) decanoic acid were dissolved in a mixture of 12 mL of squalane and 13 mL of benzyl ether. The resulting deep red solution was degassed at 65 °C reaching 15–20 μbar vacuum after 1.5 h. After switching to a N_2_ flow, the temperature of the mixture was increased to 200 °C at a rate of 5 °C min^−1^ and kept at 200 °C for 1.5 h. After, the reaction temperature was increased to 300 °C at a rate of 7.5 °C min^−1^ keeping the mixture under reflux for 1 h. The nitrogen flow rate was fixed to intermediate flow (40–80 bubbles per min). The flask was then cooled down to room temperature under an inert atmosphere. The black colloidal solution was washed 2 times with an excess amount of acetone (45 mL) and centrifuged at 4500 rpm for 20 min to collect the particle as a black precipitate. The final particles were dispersed in chloroform (8 mL) for further experiments.

### Synthesis of Co_*x*_Fe_3−*x*_O_4_ nanocubes

Cobalt ferrite NCs (Co_0.5_Fe_2.5_O_4_) were synthesized by thermal decomposition following an established procedure, with suitable modifications.^16^ The protocol relies on the synthesis of particles of 12 nm in size with 0.5 Co stoichiometry. In a 100 mL three-neck flask connected to a standard Schlenk line, 0.5 mmol (120 mg) Co(acac)_2_, 1 mmol (353 mg) Fe(acac)_3_ and 6 mmol (1033 mg) decanoic acid were dissolved in a mixture of 12 mL of squalane and 13 mL of benzyl ether. The resulting deep red solution was degassed at 65 °C for 2 h reaching 16–20 μbar vacuum after 2 h. After switching to a N_2_ flow, the temperature of the mixture was increased to 200 °C at a rate of 5 °C min^−1^ and kept at 200 °C for 1.5 h. After, the reaction temperature was increased to 300 °C at a rate of 7.5 °C min^−1^ keeping the mixture under reflux for 1 h. The nitrogen flow rate was fixed to intermediate flow (5–120 bubbles per min). The flask was then cooled down to room temperature under an inert atmosphere. The black colloidal solution was washed 2 times with an excess amount of acetone (45 mL) and centrifuged at 4500 rpm for 20 min to collect the particle as a black precipitate. The final particles were dispersed in chloroform (8 mL) for further experiments.

### Synthesis of Zn_*x*_Fe_3−*x*_O_4_ nanocubes

Zinc ferrite NCs (Zn_*x*_Fe_3−*x*_O_4_ NCs) were synthesized by thermal decomposition. The protocol here reported relies on the synthesis of particles of 12 nm in size and with 0.2 Zn stoichiometry. In a 100 mL three-neck flask connected to a standard Schlenk line, 0.5 mmol (131 mg) Zn(acac)_2_, 1 mmol (353 mg) Fe(acac)_3_ and 7 mmol (1033 mg) decanoic acid were dissolved in a mixture of 12 mL of squalane and 13 mL of benzyl ether. The solution was degassed at 65 °C for 1.5 h reaching 16–20 μbar vacuum after 1.5 h. After switching to a N_2_ flow, the temperature of the mixture was increased to 200 °C at a rate of 5 °C min^−1^ and was then kept at 200 °C for 2 h. The reaction temperature was increased to 300 °C at a rate of 7.5 °C min^−1^ keeping the mixture refluxing for 1 h. The nitrogen flow rate was fixed to intermediate flow (80 bubbles per min). The flask was left to room temperature to cooled down under an inert atmosphere. The black colloidal solution was washed 2 times with an excess amount of acetone (45 mL) and centrifuged at 4500 rpm for 20 min to collect the particles as a black precipitate. The final particles were dispersed in chloroform (8 mL) for further experiments.

### Syntheses of Zn_*x*_Co_*y*_Fe_3-(*x*+*y*)_O_4_ ferrite nanocubes

Zinc–cobalt NCs (Zn_0.1_Co_0.2_Fe_2.7_O_4_ NCs) of 15 nm were synthesized using the here detailed protocol: in a 100 mL three-neck flask connected to a standard Schlenk line, 0.25 mmol (63 mg) Zn(acac)_2_, 0.25 mmol (62 mg) Co(acac)_2_, 1 mmol (353 mg) Fe(acac)_3_ and 6 mmol (1033 mg) decanoic acid were dissolved in a mixture of 12 mL of squalane and 13 mL of benzyl ether. The solution was degassed at 65 °C for 1.5 h reaching 16–20 μbar vacuum after 1.5 h. After switching to a N_2_ flow, the temperature of the mixture was increased to 200 °C at a rate of 5 °C min^−1^ kept at 200 °C for 2 h. The reaction temperature was increased to 300 °C at a rate of 7.5 °C min^−1^ keeping the mixture refluxing for 1 h. The nitrogen flow rate was fixed to intermediate flow (80 bubbles per min). The flask was than cooled down to RT under an inert atmosphere. The black colloidal solution was washed 2 times with an excess amount of acetone (45 mL) and centrifuged at 4500 rpm for 20 min to collect the particles as a black precipitate. The final particles were dispersed in chloroform (8 mL) for further experiments.

### Phase transfer of the NCs in water with poly(maleic anhydride-*alt*-1-octadecene)

The synthesized, hydrophobic NCs dispersed in chloroform were transferred to water, using poly(maleic anhydride-*alt*-1-octadecene) (PMAO), following a protocol developed by our group with suitable modifications.^49^ NCs of 15 nm in size at a concentration of about 400 nM (were diluted with an excess of chloroform (70 mL) and sonicated for 5 minutes. A certain amount of PMAO polymer solution in chloroform (137 mM, concentration referring to the monomer units) was added, with a ratio of 500 molecules of monomer unit per nm^2^ of the NC surface. The solvent was evaporated using a Rotavapor (140 rpm, 50 °C) following three steps of evaporation under reduced pressure: 800 mbar for 30 minutes, 700 mbar for 30 minutes and finally 600 mbar until complete solvent evaporation was achieved. The dried sample was then soaked in 20 mL of borate buffer and sonicated at 65 °C for 1 h until complete solubilization was verified. The water soluble NCs were concentrated by an ultrafiltration system. The sample volume was reduced to nearly 3 mL under continuous shaking at 150 rpm for 15 min at RT. Subsequently, the sample was loaded on top of a sucrose gradient (2 mL of 20%, 3 mL of 40% and 3 mL of 60% in an ultra-centrifugal tube, from top to bottom) and centrifuged at 20 000 rpm for 45 min. The excess of polymer (visible under an UV lamp) found at the top of the sucrose gradient at 20% was removed, by collecting it with a syringe. Instead, the NCs, usually found in the 40% to 60% fraction of the sucrose gradient, were collected and further washed with pure water (Milli-Q) three times to remove the excess of sucrose and finally concentrated to a final volume of about 4 mL in water.

### Poly(ethylene glycol) functionalization

The functionalization of the polymeric shell of NCs with diamine–PEG (NH_2_–PEG–NH_2_) (MW 2000) was carried out following a procedure developed in our group, with minor modifications.^62^ A solution of Co-ferrite NCs of 0.1 μM was mixed with the same volume of 0.125 M 1-ethyl-3-(3-dimethylaminopropyl)carbodiimide hydrochloride (EDC) and a diamine–PEG (NH_2_–PEG–NH_2_) 0.1 mM solution in pure water. The amount of PEG was kept as 1000-fold excess compared to Co-ferrite NC molar concentration. The mixture was vigorously shaken for one minute and left under gentle shaking at 150 rpm for 2 h at RT. Finally, the solution was diluted 3 times to quench the reaction and washed in pure water using centrifugal filters. The final sample was collected in few microliters of water.

### Elemental analysis

Inductively coupled plasma optical emission spectroscopy (ICP-OES) analysis was performed on an iCAP 6000 spectrometer (Thermo Scientific) to quantify the concentration of the elements of interest. Briefly, 25 μL samples were digested in 2.5 mL of aqua regia (HCl : HNO_3_ ratio is 3 : 1 (v/v)) overnight and diluted with 25 mL of Milli-Q water prior to the measurement.

### Transmission electron microscopy

Overview transmission electron microscopy (TEM) analyses were carried out on a JEOL JEM-1011 instrument, using an acceleration voltage of 100 kV. The sample preparation was conducted by drop-casting a droplet of the sample solution onto a 400 mesh ultra-thin carbon-coated copper grid with a subsequent removal of the solvent by evaporation at RT. For high-resolution TEM and compositional analyses, an image-C_S_-corrected JEOL JEM-2200FS, operated at 200 kV, was used. Energy-dispersive X-ray spectroscopy (EDS) analyses were carried out using a Bruker XFlash 5060 silicon-drift detector. For these analyses, a small volume of each sample was drop-cast onto an ultrathin carbon/holey carbon-coated Au grid. STEM-EDS elemental maps shown here are obtained by integration of the L_α_ peak for Zn, K_α_ peak for Fe and K_β_ peak for Co in the raw spectra. Oxygen was not mapped nor quantified by STEM-EDS.

### X-Ray diffraction analysis

XRD patterns were recorded on a Malvern PANalytical Empyrean diffractometer machine. The X-ray source is operated at 40 kV and 150 mA. The diffractometer was equipped with a Cu source and a Göbel mirror to obtain a parallel beam as well as to suppress Cu Kβ radiation (1.392 Å). The 2-theta/omega scan geometry was used to acquire the data. The samples were prepared by drop casting concentrated NC solutions onto a zero background silicon substrate. The PDXL software of Rigaku was used for phase identification.

### Magnetic characterization

Magnetic measurements were performed on a superconducting quantum interference device (SQUID) from Quantum Design Inc. Hysteresis loops were recorded within the magnetic field of ±4800 kA m^−1^ at 5 K and 298 K. Samples were prepared by dropping 50 μL of solution (*ca.* 1 g L^−1^) on a Teflon tape. Magnetization curves were normalized by the metal oxide mass obtained by ICP.

### AC magnetic measurements

The inductive magnetic characterization was carried out with a home-made AC magnetometer developed by Advanced Magnetic Instrumentation Unit at iMdea Nanociencia. AC hysteresis loops were recorded at different field intensities (8–16–24 kA m^−1^) and frequencies (100–300 kHz). Each AC hysteresis loop measurement is obtained as the average of three repetition of AC magnetization curves under given field conditions, leading to an averaged and standard deviation of the magnetic area values, among other magnetic parameters related to the acquired AC hysteresis loop. The experiments were performed by using a 40 μL volume of the different ferrite NC colloidal samples at a concentration of 2 g of metal elements (Co + Fe or Zn + Fe or Zn + Co + Fe, for Co-ferrite, Zn-ferrite and Zn-Co-ferrite respectively) per liter, when changing media viscosity by adding glycerol at 0%–15%–36%–60%–80% (v/v) fraction in a water volume. The AC magnetization signal was normalized to the mass of magnetic elements present in the studied magnetic colloid.

### Calorimetric measurements of the specific absorption rate

A commercially available magnetic nano-heating device (DM100 Series, nanoScale Biomagnetics) was used to evaluate the heating performance (SAR values) under quasi-adiabatic conditions. The water soluble NCs were exposed to an alternating magnetic field (from 12 up to 32 kA m^−1^) at 2 different frequencies (105, 300 kHz). All measurements were performed using 50 μL of sample in water at a total metal concentration of 2 g L^−1^ (Co + Fe or Zn + Fe or Zn + Co + Fe, for Co-ferrite, Zn-ferrite and Zn-Co-ferrite, respectively) and the SAR values were normalized using the metal mass concentration determined by elemental analysis. SAR values were calculated according to the following equation:1
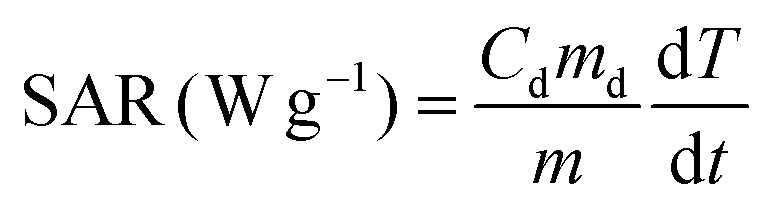
where *C*_d_ is the specific heat capacity of the dispersion medium (for water *C*_d_ = 4185 J g^−1^ K^−1^), *m*_d_ is the dispersion medium mass, and *m* is defined as the total metal mass per g of the dispersion, and d*T*/d*t* is the initial slope of the temperature enhancement *versus* time. To calculate the parameter d*T*/d*t*, temperature data points collected within the first 60 seconds were used to obtain the slope of the curve deriving from the linear fitting of these points. Each data point is the average d*T*/d*t* value of three independent measurements.

### Relaxivity measurements

Water solutions of NCs containing different magnetic material concentrations ranging from 0.001 to 1 mM (0.001–0.005–0.01–0.05–0.1–0.2–0.4–0.6–0.8–1 mM) were prepared. The longitudinal (*T*_1_) and transverse (*T*_2_) relaxation times were measured at 40 °C using a Minispec spectrometer (Bruker, Germany) mq 20–0.5 T (400 kA m^−1^) and mq 60–1.5 T (1200 kA m^−1^). The *T*_1_ relaxation profile was obtained using an inversion-recovery sequence, with 20 data points and 4 acquisitions for each measurement. The *T*_2_ relaxation time was measured using a Carr–Purcell–Meiboom–Gill (CPMG) spin-echo pulse sequence with 200 data points with an interecho time of 0.5 ms. The relaxivities *r*_*i*_ (*i* = 1, 2) were determined by the following equation:
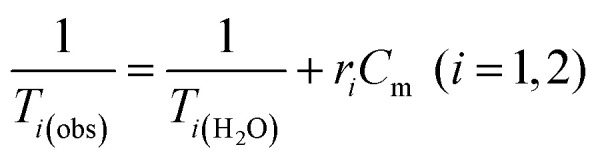
where *C*_m_ is the concentration of magnetic ions. The values are reproducible within 5% deviation.

### MPI relaxometry

MPI analyses were performed in a custom made MPI relaxometer device.^7^ Representative samples of ferrite NCs were analyzed as suspensions in chloroform or water of 500 μL at a fixed iron concentration of 1.0 mg_Fe_ mL^−1^; the samples were sonicated before performing each MPI relaxometry measurement. All MPI studies were conducted under a magnetic excitation field strength and frequency of 16 kA m^−1^ and 16.8 kHz, respectively. The MPI signals generated were normalized to the MPI signal of a commercial MPI tracer, VivoTrax™ (Resorvist® licensed for distribution in USA through Magnetic Insight for pre-clinical MPI studies) (500 μL, 1.0 mg_Fe_ mL^−1^).

### Cell culture

Mouse cerebral endothelial cells (bEnd3) were cultured in Dulbecco's Modified Eagle's Medium (DMEM) supplemented with 10% fetal bovine serum (FBS), 1% penicillin streptomycin (PS), 1% pyruvate, 1% non-essential amino acids (100×)(MEM) and 1% glutamine at 37 °C, 5% CO_2_ and 95% humidity. Cells were split every 3–4 days, before reaching 100% confluence. Mouse glioblastoma cells (GL261) were cultured in Dulbecco's Modified Eagle's Medium (DMEM) supplemented with 10% fetal bovine serum (FBS), 1% penicillin streptomycin (PS) and 1% glutamine at 37 °C, 5% CO_2_ and 95% humidity. Cells were split every 2–3 days, before reaching 100% confluence.

### NC cytotoxicity studies

Viability of the bEnd3 and GL261 cells upon incubation with NCs were tested with a dead/live assay by flow cytometry. Briefly, cells at a concentration of 10^5^ cells per well were seeded in a 24 multi-well plate and allowed to adhere for 24 h. The day after, Zn_0.2_Fe_2.8_O_4_ NCs (13 ± 1 nm) or Co_0.5_Fe_2.5_O_4_ NCs (12 ± 2 nm), diluted in fresh medium (at 100 nM, 50 nM and 25 nM NC concentration corresponding to a NC dose of 0.6, 0.3 and 0.15 mg mL^−1^ respectively), were added to the cells and incubated for additional 24 h, 48 h or 72 h at 37 °C. At each time point of interest, the cells were detached from the wells, washed several times in PBS and stained with a dead cell dye, ethidium homodimer-1 (EthD-1), following the manufacture's protocol and then analysed by flow cytometry (FACSariaIII -BD-bioscience device). Briefly, a staining solution of 2 μL of dye (EthD-1) in 1 mL of phosphate buffered saline (PBS) was prepared. Upon removal of media, cells in each well were incubated with 500 μL of the staining solution for 20 min at RT. Next, cells were rinsed twice with PBS solution and then collected in 200 μL of PBS for the analysis with FACS to count the dead cells. Cells with compromised cellular membranes allowed the dye to enter inside the cell, thus identifying as dead cells. In contrast, non-stained cells corresponded to viable cells. Phase contrast imaging of the cells treated with the different NCs were performed using an optical microscope (Motic AE31, 20× magnification) confirming the results obtained by FACS. The two samples (Zn_0.2_Fe_2.8_O_4_ NCs and Co_0.5_Fe_2.5_O_4_ NCs) were sterilized before the use; briefly, they were placed in open vials inside a closed biological fume hood and exposed to three cycle of UV light (20 minutes each).

## Author contributions

T.P. and N.S. conceived and designed the study. N.S. produced and characterized the materials. N.S. and H.G. performed the calorimetric measurements. P.G performed the SQUID magnetic characterization and the XRD analysis of the samples. R.B. performed the high resolution TEM and the EDS analysis. A.C.S performed the MPI relaxivity measurements. N.S. and F.J.T performed the AC magnetic measurements. N.S. and S.F. performed *in vitro* experiments. T.P., N.S. and H.G. wrote the paper with contributions obtained from all authors.

## Conflicts of interest

The authors declare no competing financial interests.

## Supplementary Material

NR-013-D1NR01044A-s001

## References

[cit1] Guardia P., Riedinger A., Nitti S., Pugliese G., Marras S., Genovese A., Materia M. E., Lefevre C., Manna L., Pellegrino T. (2014). J. Mater. Chem. B.

[cit2] Song Q., Zhang Z. J. (2012). J. Am. Chem. Soc..

[cit3] Anselmo A. C., Mitragotri S. (2015). AAPS J..

[cit4] Allione M., Torre B., Casu A., Falqui A., Piacenza P., Di Corato R., Pellegrino T., Diaspro A. (2011). J. Appl. Phys..

[cit5] Martinez-boubeta C., Simeonidis K., Makridis A., Angelakeris M., Serantes D., Baldomir D., Saghi Z., Midgley P. A. (2013). Sci. Rep..

[cit6] Materia M. E., Guardia P., Sathya A., Pernia Leal M., Marotta R., Di Corato R., Pellegrino T. (2015). Langmuir.

[cit7] Bauer L. M., Situ S. F., Griswold M. A., Samia A. C. S. (2016). Nanoscale.

[cit8] Périgo E. A., Hemery G., Sandre O., Ortega D., Garaio E., Plazaola F., Teran F. J. (2015). Appl. Phys. Rev..

[cit9] Guardia P., Di Corato R., Lartigue L., Wilhelm C., Espinosa A., Garcia-Hernandez M., Gazeau F., Manna L., Pellegrino T. (2012). ACS Nano.

[cit10] Cristina A., Samia S. (2016). Prog. Nat. Sci.: Mater. Int..

[cit11] Carta D., Casula M. F., Falqui A., Loche D., Mountjoy G., Sangregorio C., Corrias A. (2009). J. Phys. Chem. C.

[cit12] Xu Y., Sherwood J., Qin Y., Holler R. A., Bao Y. (2015). Nanoscale.

[cit13] Yang Y., Liu X., Yang Y., Xiao W., Li Z., Xue D., Li F., Ding J. (2013). J. Mater. Chem. C.

[cit14] Berkowitz A. E., Schuele W. J. (1959). J. Appl. Phys..

[cit15] Lee J.-G., Park J. Y., Oh Y.-J., Kim C. S. (1998). J. Appl. Phys..

[cit16] Sathya A., Guardia P., Brescia R., Silvestri N., Pugliese G., Nitti S., Manna L., Pellegrino T. (2016). Chem. Mater..

[cit17] Noh S. H., Na W., Jang J. T., Lee J. H., Lee E. J., Moon S. H., Lim Y., Shin J. S., Cheon J. (2012). Nano Lett..

[cit18] Cabrera D., Lak A., Yoshida T., Materia M. E., Ortega D., Ludwig F., Guardia P., Sathya A., Pellegrino T., Teran F. J. (2017). Nanoscale.

[cit19] Horev-Azaria L., Baldi G., Beno D., Bonacchi D., Golla-Schindler U., Kirkpatrick J. C., Kolle S., Landsiedel R., Maimon O., Marche P. N., Ponti J., Romano R., Rossi F., Sommer D., Uboldi C., Unger R. E., Villiers C., Korenstein R. (2013). Part. Fibre Toxicol..

[cit20] Balakrishnan P. B., Silvestri N., Fernandez-Cabada T., Marinaro F., Fernandes S., Fiorito S., Miscuglio M., Serantes D., Ruta S., Livesey K., Hovorka O., Chantrell R., Pellegrino T. (2020). Adv. Mater..

[cit21] Goldhaber S. B. (2003). Regul. Toxicol. Pharmacol..

[cit22] Ammar S., Jouini N., Fiévet F., Beji Z., Smiri L., Moliné P., Danot M., Grenèche J.-M. (2006). J. Phys.: Condens. Matter.

[cit23] Chinnasamy C. N., Narayanasamy A., Ponpandian N., Chattopadhyay K., Guerault H., Greneche J. M. (2000). J. Phys.: Condens. Matter.

[cit24] Naseri M. G., Saion E. B., Hashim M., Shaari A. H., Ahangar H. A. (2011). Solid State Commun..

[cit25] Bárcena C., Sra A. K., Chaubey G. S., Khemtong C., Liu J. P., Gao J. (2008). Chem. Commun..

[cit26] Chen Z. P., Fang W. Q., Zhang B., Yang H. G. (2013). J. Alloys Compd..

[cit27] Wan J., Jiang X., Li H., Chen K. (2012). J. Mater. Chem..

[cit28] Salazar-Alvarez G., Qin J., Šepelák V., Bergmann I., Vasilakaki M., Trohidou K. N., Ardisson J. D., Macedo W. A. A., Mikhaylova M., Muhammed M., Baró M. D., Nogués J. (2008). J. Am. Chem. Soc..

[cit29] Köseoğlu Y., Baykal A., Gözüak F., Kavas H. (2009). Polyhedron.

[cit30] Arulmurugan R., Jeyadevan B., Vaidyanathan G., Sendhilnathan S. (2005). J. Magn. Magn. Mater..

[cit31] Köseoğlu Y., Alan F., Tan M., Yilgin R., Öztürk M. (2012). Ceram. Int..

[cit32] Paulsen J. A., Ring A. P., Lo C. C. H., Snyder J. E., Jiles D. C. (2005). J. Appl. Phys..

[cit33] Vaidyanathan G., Sendhilnathan S. (2008). Physica B: Condens. Matter.

[cit34] Sanpo N., Berndt C. C., Wen C., Wang J. (2013). Acta Biomater..

[cit35] Gharibshahian M., Nourbakhsh M. S., Mirzaee O. (2018). J. Sol-Gel Sci. Technol..

[cit36] Ben Ali M., El Maalam K., El Moussaoui H., Mounkachi O., Hamedoun M., Masrour R., Hlil E. K., Benyoussef A. (2016). J. Magn. Magn. Mater..

[cit37] SINGHAL S., JAUHAR S., CHANDRA K., BANSAL S. (2013). Bull. Mater. Sci..

[cit38] Moyet R. P., Cardona Y., Vargas P., Silva J., Uwakweh O. N. C. (2010). Mater. Charact..

[cit39] Wang T., Luan Z.-Z., Ge J.-Y., Liu L., Wu D., Lv Z.-P., Zuo J.-L., Sun S. (2018). Phys. Chem. Chem. Phys..

[cit40] Mameli V., Musinu A., Ardu A., Ennas G., Peddis D., Niznansky D., Sangregorio C., Innocenti C., Thanh N. T. K., Cannas C. (2016). Nanoscale.

[cit41] Sathya A., Guardia P., Brescia R., Silvestri N., Pugliese G., Nitti S., Manna L., Pellegrino T. (2016). Chem. Mater..

[cit42] Gilbert K. E., Gajewski J. J. (1982). J. Org. Chem..

[cit43] Lak A., Kahmann T., Schaper S. J., Obel J., Ludwig F., Müller-Buschbaum P., Lipfert J. (2019). ACS Appl. Mater. Interfaces.

[cit44] Ma Y. Q., Pang Y. Y. (2015). Chin. J. Polym. Sci..

[cit45] Najafabadi A. T., Khodadadi A. A., Parnian M. J., Mortazavi Y. (2016). Appl. Catal., A.

[cit46] Bao Y., An W., Turner C. H., Krishnan K. M. (2010). Langmuir.

[cit47] Pardo A., Yáñez S., Piñeiro Y., Iglesias-Rey R., Al-Modlej A., Barbosa S., Rivas J., Taboada P. (2020). ACS Appl. Mater. Interfaces.

[cit48] López-Ortega A., Estrader M., Salazar-Alvarez G., Estradé S., Golosovsky I. V., Dumas R. K., Keavney D. J., Vasilakaki M., Trohidou K. N., Sort J., Peiró F., Suriñach S., Baró M. D., Nogués J. (2012). Nanoscale.

[cit49] Di Corato R., Quarta A., Piacenza P., Ragusa A., Figuerola A., Buonsanti R., Cingolani R., Manna L., Pellegrino T. (2008). J. Mater. Chem..

[cit50] Mehdaoui B., Carrey J., Stadler M., Cornejo A., Nayral C., Delpech F., Chaudret B., Respaud M. (2012). Appl. Phys. Lett..

[cit51] Hergt R., Dutz S. (2007). J. Magn. Magn. Mater..

[cit52] Gleich B., Weizenecker J. (2005). Nature.

[cit53] Song G., Kenney M., Chen Y. S., Zheng X., Deng Y., Chen Z., Wang S. X., Gambhir S. S., Dai H., Rao J. (2020). Nat. Biomed. Eng..

[cit54] Wang Q., Ma X., Liao H., Liang Z., Li F., Tian J., Ling D. (2020). ACS Nano.

[cit55] Du Y., Liu X., Liang Q., Liang X. J., Tian J. (2019). Nano Lett..

[cit56] Arami H., Ferguson R. M., Khandhar A. P., Krishnan K. M. (2013). Med. Phys..

[cit57] Abenojar E. C., Wickramasinghe S., Bas-Concepcion J., Samia A. C. S. (2016). Prog. Nat. Sci.: Mater. Int..

[cit58] Torres T. E., Lima E., Calatayud M. P., Sanz B., Ibarra A., Fernández-Pacheco R., Mayoral A., Marquina C., Ibarra M. R., Goya G. F. (2019). Sci. Rep..

[cit59] Jun Y. W., Huh Y. M., Choi J. S., Lee J. H., Song H. T., Kim S., Yoon S., Kim K. S., Shin J. S., Suh J. S., Cheon J. (2005). J. Am. Chem. Soc..

[cit60] Vuong Q. L., Berret J. F., Fresnais J., Gossuin Y., Sandre O. (2012). Adv. Healthcare Mater..

[cit61] Duan H., Kuang M., Wang X., Wang Y. A., Mao H., Nie S. (2008). J. Phys. Chem. C.

[cit62] Sperling R. A., Pellegrino T., Li J. K., Chang W. H., Parak W. J. (2006). Adv. Funct. Mater..

[cit63] Newcomb E. W. and Zagzag D., in CNS Cancer, Humana Press, Totowa, NJ, 2009, pp. 227–241

[cit64] Li G., Simon M. J., Cancel L. M., Shi Z.-D., Ji X., Tarbell J. M., Morrison B., Fu B. M. (2010). Ann. Biomed. Eng..

[cit65] Martínez-Rodríguez N. L., Tavárez S., González-Sánchez Z. I. (2019). Toxicol. In Vitro.

[cit66] Alhadlaq H. A., Akhtar M. J., Ahamed M. (2015). Cell Biosci..

[cit67] Rivero M., Marín-Barba M., Gutiérrez L., Lozano-Velasco E., Wheeler G. N., Sánchez-Marcos J., Muñoz-Bonilla A., Morris C. J., Ruiz A. (2019). J. Nanopart. Res..

[cit68] Saquib Q., Al-Khedhairy A. A., Ahmad J., Siddiqui M. A., Dwivedi S., Khan S. T. and Musarrat J., 10.1016/j.taap.2013.09.001

