# Supplementary material for: Di- and tri-component spinel ferrite nanocubes: synthesis and their comparative characterization for theranostic applications
Source: Nanoscale. 2021 Aug 3;13(32):13665–80. doi: 10.1039/d1nr01044a (PMC8374679; doi:10.1039/d1nr01044a)
Supplement: NR-013-D1NR01044A-s001 [file NR-013-D1NR01044A-s001.pdf]

Electronic Supplementary Material (ESI) for Nanoscale.
This journal is © The Royal Society of Chemistry 2021

Electronic Supplementary Information

Di- and tri-component spinel ferrite nanocubes: synthesis and their comparative characterization for theranostic applications

Niccolò Silvestri[1], Helena Gavilan Rubio[1], Pablo Guardia[1,2], Rosaria Brescia[1], Soraia Fernandes[1°], Anna Cristina S. Samia[3], Francisco J. Teran[4,5] and Teresa Pellegrino[1*].

1. Istituto Italiano di Tecnologia, Via Morego 30, 16163 Genova, Italy.
2. IREC-Catalonia Institute for Energy Research- Jardins de les Dones de Negre 1, Sant Adria de Besos, 08930 Barcelona, Spain.
3. Department of Chemistry, Case Western Reserve University, 10900 Euclid Avenue, Cleveland, OH 44106, USA
4. iMdea Nanociencia, Campus Universitario de Cantoblanco, 28049 Madrid, Spain.
5. Nanobiotecnología (iMdea-Nanociencia), Unidad Asociada al Centro Nacional de Biotecnología (CSIC), 28049 Madrid, Spain.

°current affiliation: International Clinical Research Center (FNUSA-ICRC), St. Anne's University

Hospital, Brno, Czech Republic

* corresponding author: Teresa.Pellegrino@iit.it

Keywords: ***ferrites, nanocubes, magnetic hyperthermia, magnetic particle imaging, contrast agents, tracers***

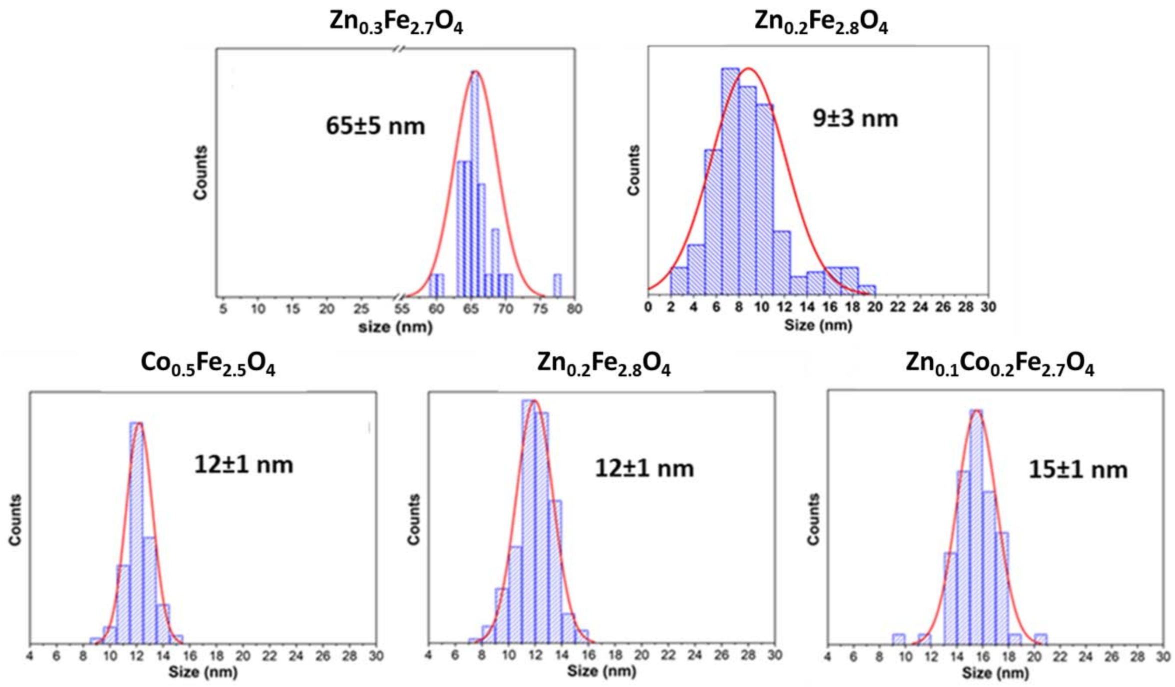

Figure S1. *Cube-edge length TEM size distributions of the as-synthesized ferrite nanocubes samples reported in Figure 2: 65 ± 5 nm for* $Zn_{0.3}Fe_{2.7}O_4$ *, 9 ± 3 nm for* $Zn_{0.2}Fe_{2.8}O_4$*, 12 ± 1 nm for* $Co_{0.5}Fe_{2.5}O_4$ *, 12 ± 1 nm for* $Zn_{0.2}Fe_{2.8}O_4$*, and 15 ± 1nm for the* $Zn_{0.1}$ $Co_{0.2}Fe_{2.7}O_4$ *samples, respectively.*

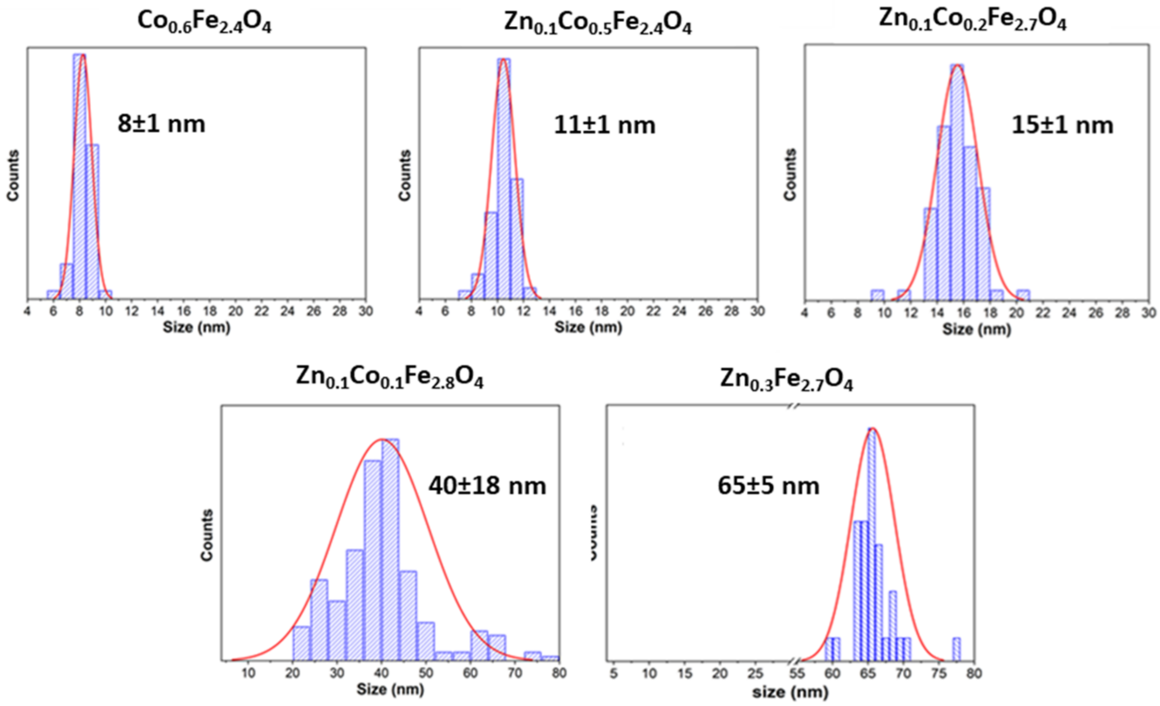

Figure S2. *Cube-edge length TEM size distributions of the as-synthesized Zn-Co-ferrite NCs samples reported in Figure 3, which were obtained by varying the Co to Zn feed molar ratio.*

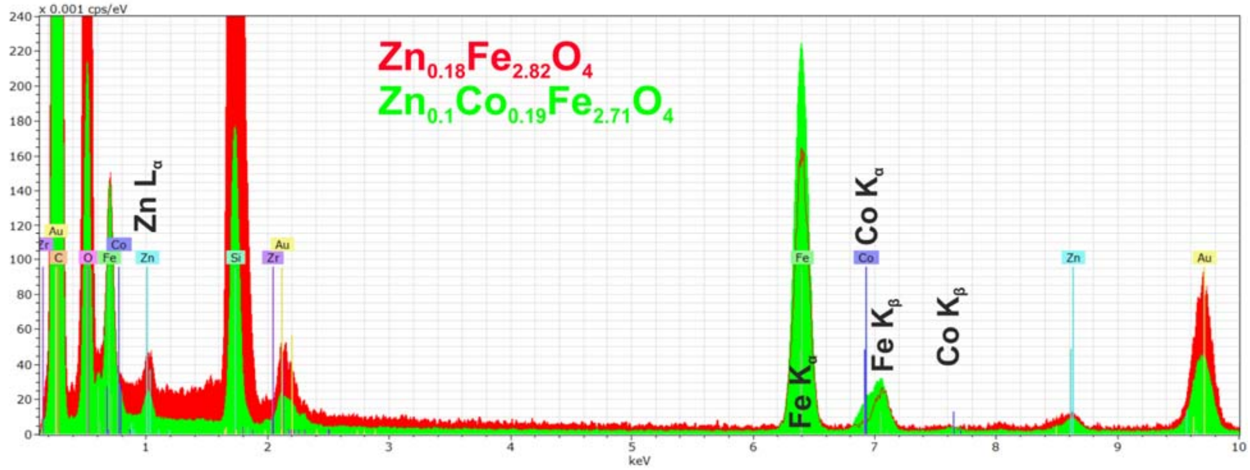

Figure S3. *Overview STEM-EDS spectra collected for 12 ± 1 nm Zn-ferrite NCs (red) and 15±1 nm Zn-Co-ferrite NCs (green). These spectra have been used for the quantification reported in Table S1.*

Table S1. *Elemental analysis by STEM-EDS obtained for the Zn-ferrite NCs with an average size of 12 ± 1 nm and for the Zn-Co-ferrite NCs with average size of 15 ± 1 nm, respectively. The cation content calculated from EDS measurements resulted into the following compositions;* $Zn_{0.2}Fe_{2.8}O_4$ *and* $Zn_{0.1}Co_{0.2}Fe_{2.7}O_4$*. These stoichiometric ratios are in close agreement with the elemental analysis results measured by ICP-AES measurements, which are* $Zn_{0.2}Fe_{2.8}O_4$ *and* $Zn_{0.1}Co_{0.2}Fe_{2.7}O_4$*, respectively.*

Sample	Element	Atomic %	Relative Error %
12±1 nm $Zn_xFe_{3-x}O_4$	Fe	95	3
	Zn	5	5
15±1 nm $Zn_xCo_yFe_{3-(x+y)}O_4$	Fe	90	3
	Co	7	4
	Zn	3	4

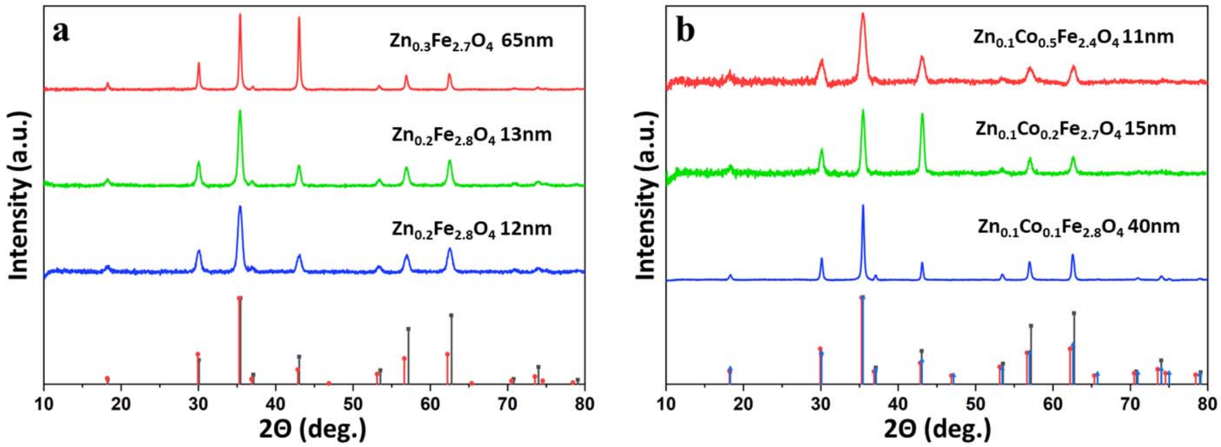

Figure S4. *Size-dependent XRD patterns of the different ferrite NCs:* ***a****) Zn-ferrite NCs and* ***b****) Zn-Co-ferrite NCs. On the lower part in grey, the peaks for the inverse spinel Fe_3O_4 powder (JCPDS card no. 00-001-1111), in red the theoretical peaks for $ZnFe_2O_4$ powder (JCPDS card no. 00-022-1012,) and in blue the theoretical peaks for $CoFe_2O_4$ powder (JCPDS card no. 00-022-1086) are represented, respectively.*

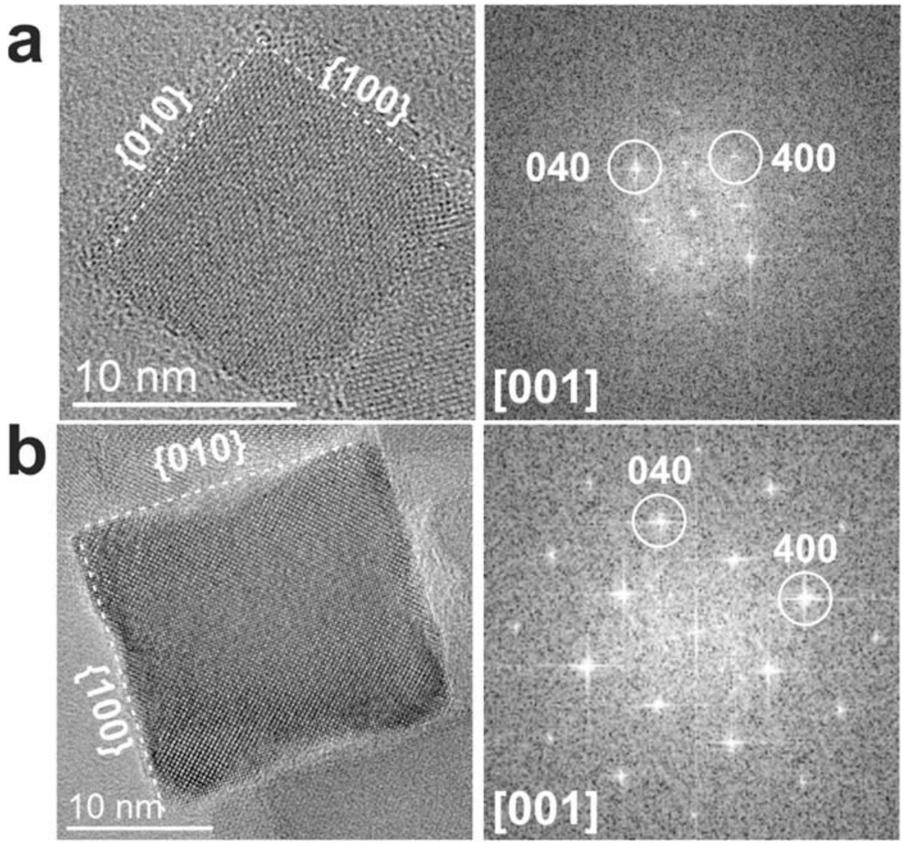

Figure S5 *HRTEM images of two samples of mixed ferrite nanocubes,* ***a****)* $Zn_{0.2}Fe_{2.8}O_4$ *and* ***b****)* $Zn_{0.1}Co_{0.2}Fe_{2.7}O_4$*, with corresponding fast Fourier transform (FFT). For both samples, the observed FFTs match with the inverse spinel* Fe_3O_4 *phase and the NCs exhibit the expected {h00} faceting.*

Table S2. *Table summarizing the average hydrodynamic size (Dh) of representative water-dispersed ferrite NC samples measured using intensity, number, and volume fittings, respectively.*

Sample	Core size (nm)	PDI	Dh by Intensity (nm)	Dh by Number (nm)	Dh by Volume (nm)
Fe_3O_4 NCs	15 ±2	0.19	54 ±24	25 ±8	34 ±15
$Co_{0.5}Fe_{2.5}O_4$ NCs	12 ±1	0.14	32 ±12	18 ±5	23 ±8
$Zn_{0.2}Fe_{2.8}O_4$ NCs	13 ±1	0.12	57 ±20	31 ±9	40 ±15
$Zn_{0.1}Co_{0.5}Fe_{2.4}O_4$ NCs	11 ±1	0.13	36 ±14	21 ±6	26 ±10

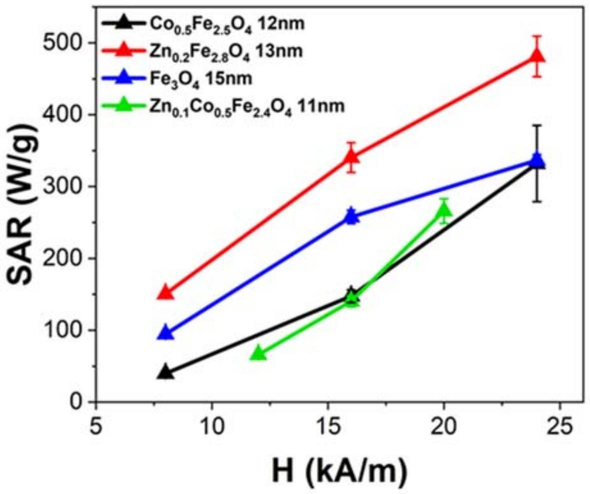

Figure S6. *SAR characterization by AC magnetometry (inductive measurement) of different ferrite NC samples in water of comparable sizes at 300 kHz and at different field conditions (8, 16, and 24 kA/m).*

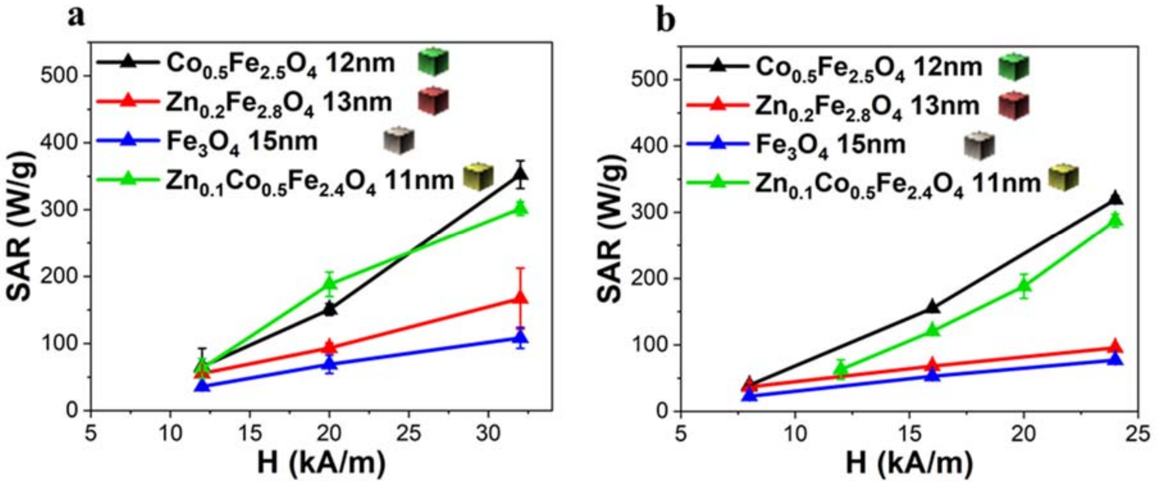

Figure S7. *Comparison of the SAR values of different ferrite NC samples obtained by inductive and calorimetric measurement methods.* ***a****) Calorimetric SAR measurements of the different ferrite nanocubes samples (2g/L, total metals concentration: Co+Fe or Zn+Fe or Zn+Co+Fe, for Co-ferrite, Zn-ferrite and Zn-Co-ferrite respectively) of comparable sizes obtained at a field frequency of 100 kHz and under 12, 20 and 32 kA/m AC magnetic field strength, respectively.* ***b****) AC magnetometry SAR measurements of the different ferrite NC samples (2g/L, total metals concentration) of comparable sizes obtained at a field frequency of 100 kHz and 8, 16. and 24 kA/m AC magnetic field strength, respectively. The parameters of field strength and frequency selected on the two instruments were selected to be as close as possible for close comparison of the results.*

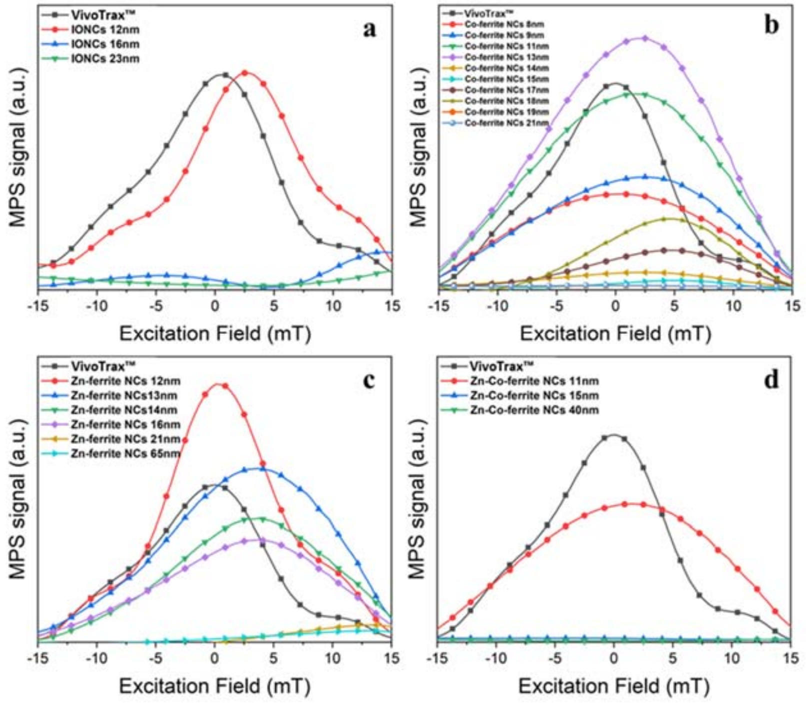

Figure S8. *MPI point spread functions (PSFs) recorded from the ferrite NC samples of varying sizes in chloroform analyzed at 16 kHz and 20 mT;* ***a****) IONCs,* ***b****) Co-ferrite NCs,* ***c****) Zn-ferrite NCs, and* ***d****) Zn-Co-ferrite NCs. The highest amplitude with the narrowest full width at half maximum were obtained for the NCs in the size range between 11 and 14 nm.*

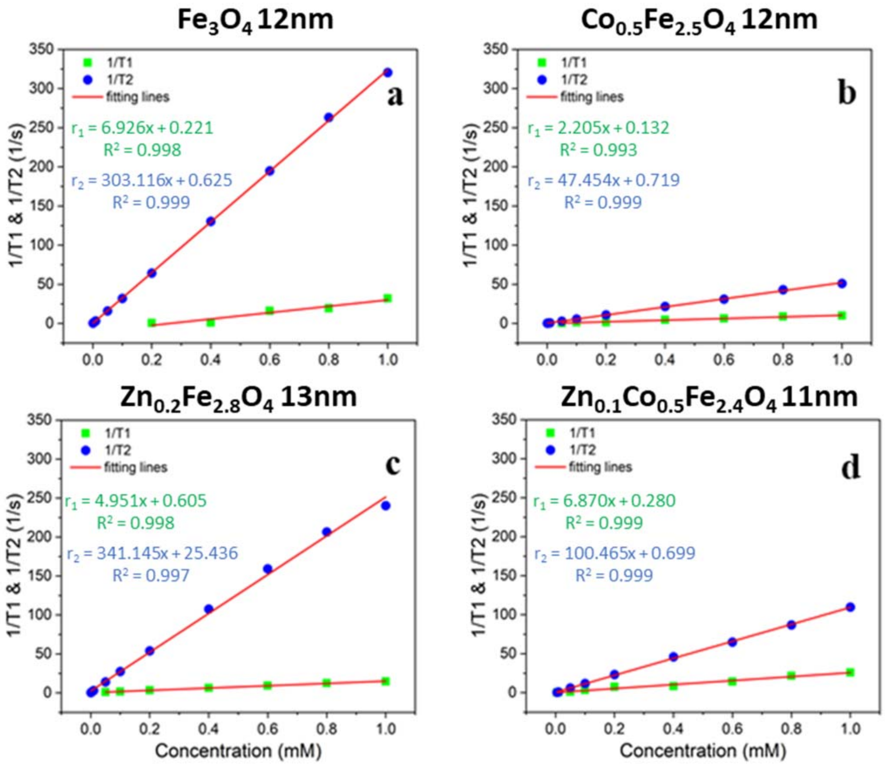

Figure S9. *Experimentally measured longitudinal and transverse relaxation rates and the corresponding linear equation fittings used to calculate regression slopes for the different ferrite NCs with varying NC concentrations obtained under 0.5 T:.* ***a****) IONCs (12nm),* ***b****) Co-ferrite NCs (12nm),* ***c****) Zn-ferrite NCs (13nm), and* ***d****) Zn-Co-ferrite NCs (11nm).*

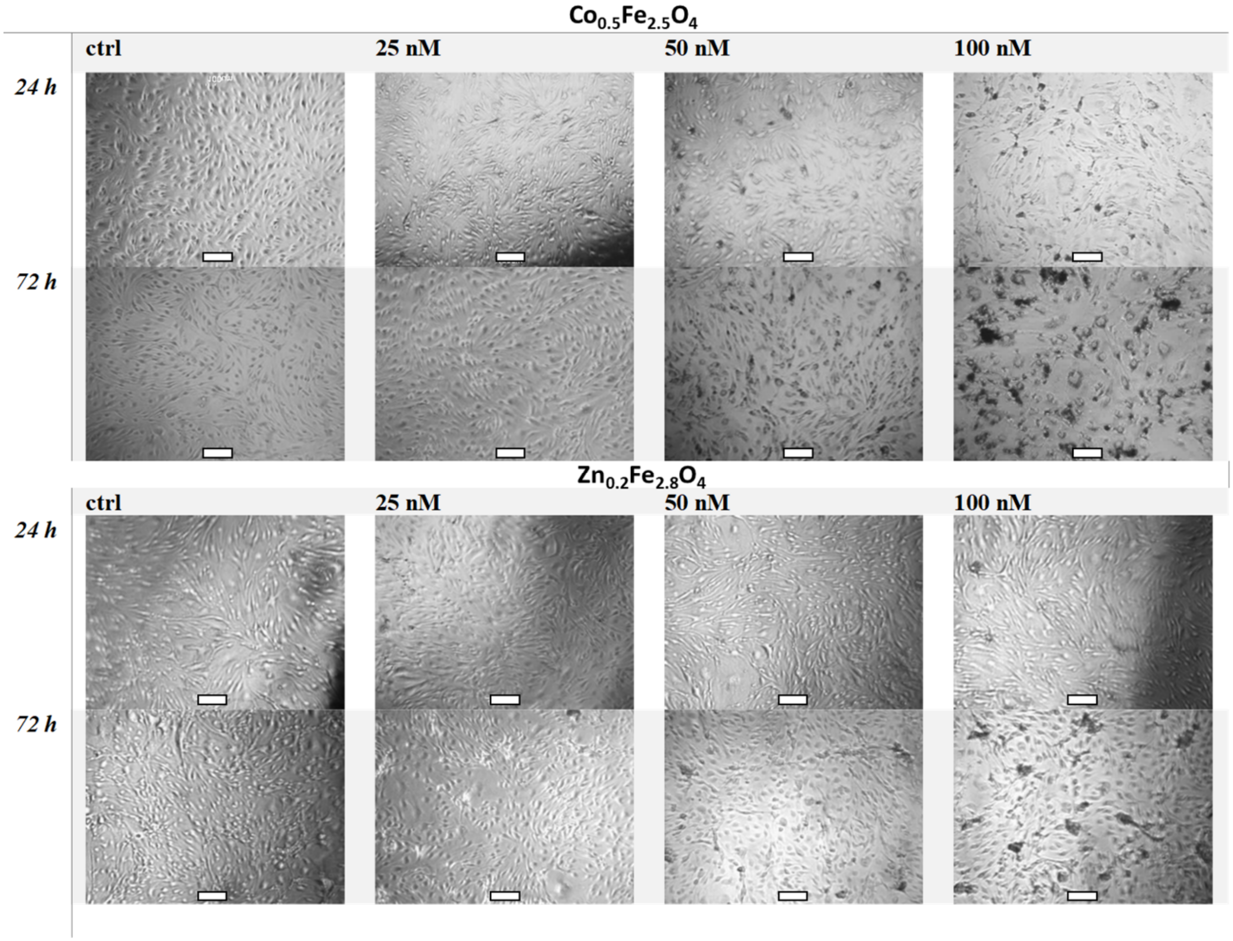

Figure S10. *Optical microscope images at (20X magnification) of bEnd.3 cells incubated with Zn-ferrite NCs (13nm) and Co-ferrite NCs (12nm) under 24 and 72 h incubation periods; using three different concentrations of NCs, 25,50 and 100nM (corresponding to 0.1, 0,2, 0,4 gFe/L and 0.02, 0.04, 0.08 gCo/L for the Co-ferrite NCs; 0.14, 0,28, 0,56 gFe/L and 0.01, 0.02, 0.04 gZn/L for the Zn-doped ferrite NCs). Scale bars indicate 100 μm in all images.*
